# Bottlebrush inspired injectable hydrogel for rapid prevention of postoperative and recurrent adhesion

**DOI:** 10.1016/j.bioactmat.2022.02.015

**Published:** 2022-02-21

**Authors:** Jushan Gao, Jinpeng Wen, Datao Hu, Kailai Liu, Yuchen Zhang, Xinxin Zhao, Ke Wang

**Affiliations:** School of Pharmacy, Health Science Center, Xi'an Jiaotong University, Xi'an, 710061, China

**Keywords:** Bottlebrush polymer, Rapid healing, Antifouling, MMT, Postoperative adhesion prevention

## Abstract

Postsurgical adhesion is a common clinic disease induced by surgical trauma, accompanying serious subsequent complications. Current non-surgical approaches of drugs treatment and biomaterial barrier administration only show limited prevention effects and couldn't effectively promote peritoneum repair. Herein, inspired by bottlebrush, a novel self-fused, antifouling, and injectable hydrogel is fabricated by the free-radical polymerization in aqueous solution between the methacrylate hyaluronic acid (HA-GMA) and N-(2-hydroxypropyl) methacrylamide (HPMA) monomer without any chemical crosslinkers, termed as H-HPMA hydrogel. The H-HPMA hydrogel can be tuned to perform excellent self-fused properties and suitable abdominal metabolism time. Intriguingly, the introduction of the ultra-hydrophilic HPMA chains to the H-HPMA hydrogel affords an unprecedented antifouling capability. The HPMA chains establish a dense hydrated layer that rapidly prevents the postsurgical adhesions and recurrent adhesions after adhesiolysis *in vivo*. The H-HPMA hydrogel can repair the peritoneal wound of the rat model within 5 days. Furthermore, an underlying mechanism study reveals that the H-HPMA hydrogel significantly regulated the mesothelial-to-mesenchymal transition (MMT) process dominated by the TGF-β-Smad2/3 signal pathway. Thus, we developed a simple, effective, and available approach to rapidly promote peritoneum regeneration and prevent peritoneal adhesion and adhesion recurrence after adhesiolysis, offering novel design ideas for developing biomaterials to prevent peritoneal adhesion.

## Highlights

• Bottlebrush inspired injectable hydrogel formed by free-radical polymerization between the methacrylate hyaluronic acid and N-(2-hydroxypropyl) methacrylamide.
• The hydrogel could rapidly prevent and heal the rat's post-surgical adhesions and recurrent adhesions after adhesiolysis within 5 days.
• The hydrogel greatly improves the antifouling capability and significantly regulates the mesothelial-to-mesenchymal transition process.

## Graphical abstract

An injectable H-HPMA hydrogel was fabricated by the free-radical polymerization in aqueous solution between the methacrylate hyaluronic acid (HA-GMA) and N-(2-hydroxypropyl) methacrylamide (HPMA) monomer without any chemical crosslinkers **(a)**. The hydrogel possesses injectability and self-fusion and anti-fouling capacity, and could rapidly prevent and heal the rat's post-surgical adhesions and recurrent adhesions after adhesiolysis within 5 days **(b)**. The underlying mechanism study reveals that the hydrogel could significantly prevent peritoneal adhesions by regulating the mesothelial-to-mesenchymal transition (MMT) process **(c)**.

**Figure:**
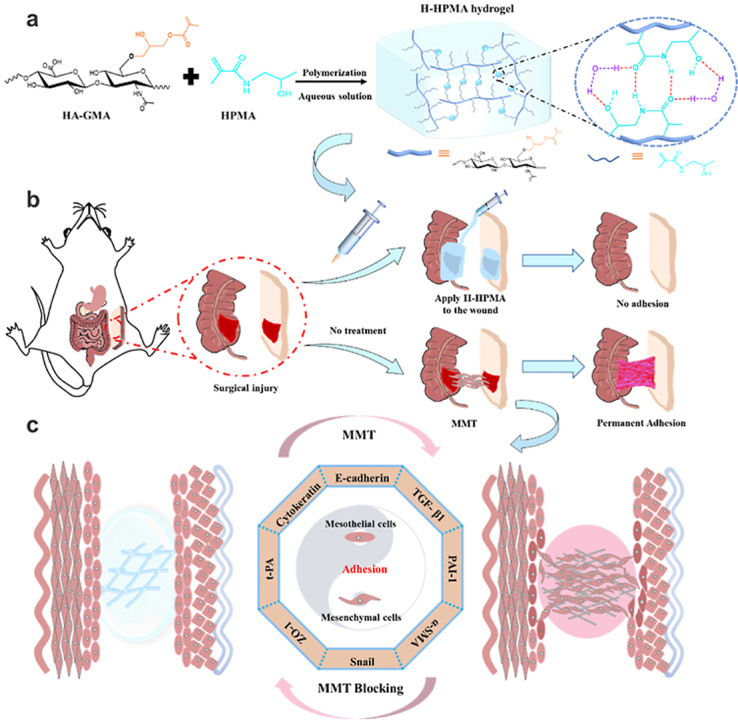

## 1 Introduction

Postsurgical peritoneal adhesion is a common clinical complication caused by the pathological connection between surgical trauma and adjacent organs or tissues, such as the injured peritoneum, omentum and serous membrane [1]. Its morbidity rate is increasing year by year and has reached over 90% for the laparotomy [2]. Generally, the peritoneal adhesion is accompanied by serious complications such as abdominal pain, diarrhea, or small bowel obstruction, considerably impeding the disease recovery and worsening the surgical failure risk [3]. The current mainstream treatments in clinics still constitute open surgery or laparoscope. However, invasive and traumatic adhesiolysis is inevitably accompanied by a high peritoneal adhesion recurrence risk, leading to treatment failure [4]. Hence, the researchers have been attempting to develop a non-surgical treatment strategy to completely prevent postsurgical peritoneal adhesion and peritoneal adhesion recurrence induced by adhesiolysis.

The peritoneum is a layer of a smooth and translucent semipermeable film in the abdominal cavity, composed primarily of mesothelial cells [[5], [6], [7]]. When the peritoneum is injured, peritoneal mesothelial cells can undergo a mesothelial-to-mesenchymal transition (MMT), gradually developing a fibroblast-like phenotype and further extruding extracellular matrix components like fibronectin, collagen I, or vascular endothelial growth factor (VEGF-α) [8,9]. These are the key triggers for post-surgical peritoneal adhesion. Clinically, the most common non-surgical approach to prevent postoperative adhesion is mainly drug treatment and biomaterial barrier administration. Nevertheless, the local or systematic drugs treatment including anti-inflammatory drugs and anti-coagulants has been proved that they would be rapidly metabolized in the peritoneal cavity, which considerably reduces their preventative effects [10]. Furthermore, to the best of our knowledge, the existing biomaterial barrier products used in clinics have demonstrated limited curative effects due to inherent defects. For example, artificial membrane barriers products such as Interceed and Seprafilm cannot completely cover irregular injured trauma and are inconvenient in practice [11,12]. The injectable polymeric solution barrier such as Adept (icodextrin solution) only has a short retention time in the local peritoneal trauma [13]. Therefore, inventing a new type of biomaterial with better performance and anti-adhesion effect is urgently required.

Recently, antifouling bottle-brush polymers have been extensively developed for the antifouling coating of *in vivo* implanted devices [[14], [15], [16]]. Among these, ultra-hydrophilic bottlebrush polymers have demonstrated excellent antifouling capability for non-specific proteins, bacteria, or fibroblasts [17,18]. Its surface-stable hydrated layer consists of highly ordered hydrogen-bonding networks and formed a dense physical barrier that could effectively prevent the adhesion of foreign substances [19]. For instance, the formed brush-like polymer using hyaluronic acid (HA) as the main chain and a highly hydrophilic polymer poly-2-methacryloyloxyethyl phosphoryl choline (PMPC) or poly-2-acrylamide-2-methylpropanesulfonic acid sodium salt (PAMPS) as the side chain can form a stable hydrated layer on the surface of the cartilage, preventing friction injury and promoting cartilage regeneration [20,21]. Moreover, the novel polymerbrush consisting of poly(l-lysine) (PLL) backbone and poly(N-(2-hydroxypropyl)methacrylamide) [poly(HPMA)] side chains has demonstrated excellent protein resistance performance [22]. Thus, these polymer brushes showed immense potential application for preventing postsurgical peritoneal adhesion. However, conventional polymer brushes serve only as thin antifouling coatings for substrates of specific shapes and have a short retention time in the abdominal cavity, thereby hindering their further application for preventing peritoneal adhesion [[23], [24], [25], [26]]. In contrast, several previous studies have shown that the injectable H-bonding hydrogel could achieve an appropriate abdominal metabolism time via a dynamically physical cross-linked network and its self-fusion could fully isolate irregular wounds, thereby preventing or alleviating postsurgical peritoneal adhesions to some extent [[27], [28], [29]]. Hence, we hypothesize that the introduction of the ultra-hydrophilic structure will make injectable H-bonding hydrogel have a similar antifouling ability, and demonstrate promising prevention effects on postsurgical peritoneal adhesion and peritoneal adhesion recurrence.

**Scheme 1:**
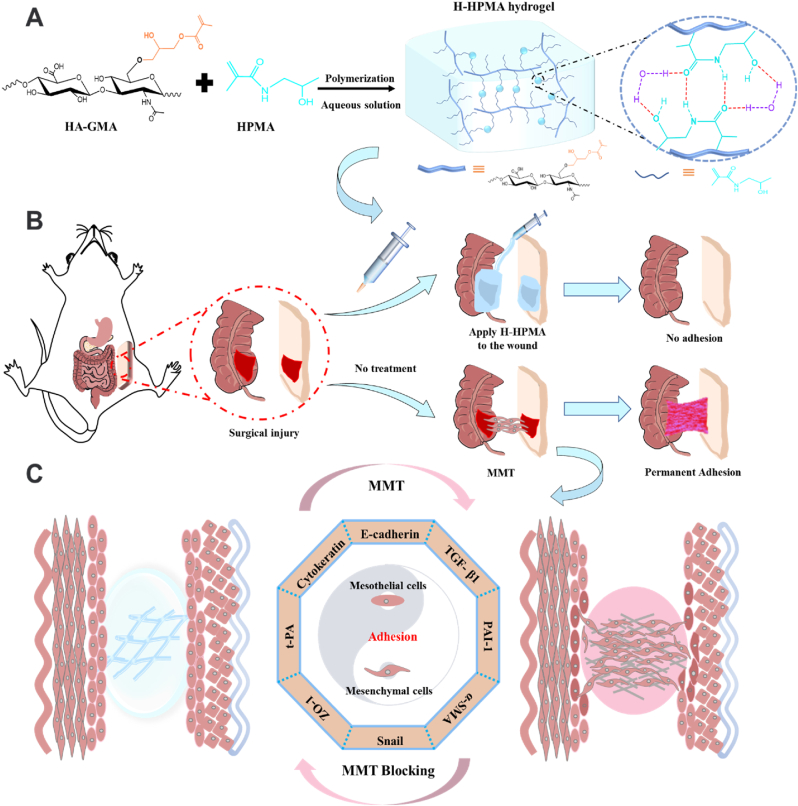
(A) Schematic illustrations of injectable H-HPMA hydrogel formation with hydrogen-bonding networks, (B) the application of H-HPMA hydrogel on a rat cecum-abdominal wall adhesion model, and (C) the underlying mechanism of H-HPMA hydrogel in post-surgical abdominal adhesions prevention.

Therefore, inspired by bottlebrush polymer, we developed a novel self-fused, antifouling, and injectable H-HPMA hydrogel by free-radical polymerization in an aqueous solution between the methacrylate hyaluronic acid (HA-GMA) and N-(2-hydroxypropyl) methacrylamide (HPMA) monomers without any crosslinker (Scheme 1A). As expected, the introduction of abundant ultra-hydrophilic HPMA chains provided the H-HPMA hydrogel with a high antifouling capability. Simultaneously, a large number of reversible hydrogen bonding networks established between HA and HPMA conferred the H-HPMA hydrogel with excellent self-fusing properties and suitable abdominal metabolism time. Meanwhile, with the mucosal repairability of the hyaluronic acid, the self-fused, antifouling and injectable H-HPMA hydrogel rapidly prevented the postsurgical peritoneal adhesions and repair the peritoneal wound within 5 days in a rat model, thereby greatly shortening the repair time of the peritoneum(Scheme 1B). Furthermore, experiments to determine the underlying mechanism revealed that the H-HPMA hydrogel significantly prevented peritoneal adhesions by regulating the mesothelial-to-mesenchymal transition (MMT) process dominated by the TGF-β-Smad2/3 signal pathway (Scheme 1C). To summarize, we developed a simple, effective and available approach to rapidly promote peritoneum regeneration and prevent peritoneal adhesion and adhesion recurrence after adhesiolysis, offering novel design ideas for developing biomaterials to prevent peritoneal adhesion.

## 2 Materials and methods

### 2.1 Materials

Hyaluronic acid sodium salt (HA, Mw:2.0 × 10^5^ Da) was purchased from the Shanghai Yuanye Biological Technology. Glycidyl methacrylate (GMA) and triethylamine (TEA) were obtained from Shanghai Macklin Biochemical Co., Ltd. Tetrabutylammonium bromide (TBAB) was obtained from Tianjin Guangfu Fine Chemical Research Institute (China). 3-aminopropylmethacrylamide hydrochloride and 2-hydroxy-4’-(2-hydroxyethoxy)-2-methylpropiophenone (IRGACURE 2959) were purchased from Sigma-Aldrich (Shanghai, China). Acryloyl chloride, glycinamide hydrochloride, amino-2-propanol, and methacrylic anhydride were supplied from Tokyo Chemical Industry Co. Ltd (Shanghai, China). All other reagents were of analytical purity. Commercial Medical Sodium Hyaluronate Gel (20173644735) was supplied by Hangzhou Xiehe Medical Supplies Company (Hangzhou, China). Micro BCA protein assay kit was purchased from Thermo Fisher Scientific Inc. (Massachusetts, USA). The monomer Fluorescein O-methacrylate was purchased from Sigma-Aldrich (St. Louis, MO, USA). HPMA was synthesized according to previous work [30]. TaqMan™ Reverse transcription Kit, SYBR Green PCR Master Mix, ELISA kit and other biological reagents above were purchased from J&L Biologica (Shanghai, China).

### 2.2 Synthesis of methacrylated hyaluronic acid

The methacrylated hyaluronic acid (HA-GMA) was synthesized as previous work by minor changes [31]. Briefly, the hydroxyl groups of hyaluronic acid sodium salt (HA) reacted with an epoxy group of glycidyl methacrylate (GMA), with the catalysis of TEA and TBAB. Firstly, hyaluronic acid is dissolved in deionized water to make a 1% HA solution. Under the magnetic stirring, TEA (140 μL) and TBAB (0.32 g) were added slowly to the solution. After 1 h, GMA (3.4 mL) was slowly dropwise added, and the reaction solution was stirred at room temperature for 24h. Finally, the resulting solution was purified by dialysis (regenerated cellulose membrane, cut-off MW 7000 Da) in deionized water for 5d and lyophilized to obtain purified HA-GMA.

### 2.3 Preparation and characterizations of H-HPMA hydrogel

#### 2.3.1 Preparation of H-HPMA hydrogel

The hydrogel precursor was formed with various mass ratios of HA-GMA and HPMA monomers (1:10, 1:5, and 2:5). Briefly, different concentrations of HA-GMA solution (0.5%, 1%, 2%, w/v) dissolved in deionized water, were mixed with HPMA monomer to form a homogeneous solution respectively. Finally, the photoinitiator IRGACURE 2959 (0.3%, w/v) was added. After the mixture homogeneously and completely dissolved, it was transferred into a syringe and polymerized for 2 h under 365 nm ultraviolet light. Then it was soaked into sterile physiological saline for 3 days to remove the impurities. The fully swollen hydrogels were sequentially squeezed through sterile needles of 18G, 20G, and 22G to form the injectable self-fused hydrogels, respectively. Obtained hydrogels were termed as H-HPMA 1:10, H-HPMA 1:5 and H-HPMA 2:5 respectively.

#### 2.3.2 Characterizations of H-HPMA hydrogel

The ^1^H NMR analysis was performed on an AVANCE III 400 MHz digital NMR spectrometer (Bruker BioSpin, Karlsruhe, Germany) to analyze the chemical structure of HA-GMA. Deuterium oxide (D_2_O) was used as a solvent with the tetramethylsilane (TMS) as the reference at room temperature. Besides, the lyophilized hydrogels samples and HA-GMA were analyzed by a Fourier transform infrared (FTIR) spectrometer (Nicolet 6700, Thermo Fisher) in the range of 500–4000 cm^−1^. The prepared H-HPMA hydrogels were fully immersed in deionized water to reach the swelling equilibrium. The Equilibrium Water Content (EWC) of the hydrogels was calculated by the gravimetric method. When the obtained hydrogels were swelled in sterile physiological saline for 3 days, the hydrogels were taken out and determined the swelling ratios by the weight difference method.

#### 2.3.3 Rheological characterization of H-HPMA hydrogel

The rheological tests of H-HPMA hydrogels after squeezing out of 22G needle were performed using an Anton-Paar rheometer (MCR 302, Austria). All rheological measurements were determined on a cone plate with a diameter of 25 mm and the temperature was set at 37 °C. In frequency sweep tests, dynamic storage modulus (G′) and loss modulus (G″) were carried out at 1% strain on each sample and the frequency range was from 0.1 to 100 Hz (corresponding to an angular frequency range from 0.628 to 628 rad/s). Dynamic time-sweep experiments were performed at a strain value of 1% and a frequency of 1 Hz over a time range from 0 to 300s. The step-strain-sweep was performed under the alternate conversion between high shear strain (500%) and low shear strain (0.5%) for three cycles under 1 Hz constant frequency (100s for each cycle). The viscosity-shear rate curves were recorded at the range from 0.01 to 1000s^−1^.

#### 2.3.4 Anti-fouling capability measurement of H-HPMA hydrogel

Nonspecific protein adsorption measurement and *in vivo* cell attachment assay were performed by using a Micro BCA protein assay kit (#23235, Thermo Fisher) and L929 cells, respectively. The nonspecific protein adsorption test was conducted as previous work [32]. When reaching swelling equilibrium, the H-HPMA 1:5 hydrogel disks with a diameter of 10 mm and a thickness of 1 mm were immersed into a 24-well plate and 1 mL BSA solution (2 mg/mL) was added. After incubation for 2 h at 37 °C, the BSA solution was removed and the hydrogel disks were rinsed three times with physiological saline to remove the protein that loosely adsorbed on the surface. Then, the BSA absorbed firmly on the hydrogel was detached after treating with 1% sodium dodecyl sulfate (SDS) solution for 2 h. The absorbed protein concentration was obtained by measuring the absorbance of 570 nm wavelength with a microplate reader (Bio-Rad, Berkeley, CA). Each test was performed in triplicate.

The fibroblasts adhesion in surgical trauma sites is the striking factor that leads to the formation of post-surgical peritoneal adhesion, so the cell attachment on the hydrogels was evaluated using L929 cells. The H-HPMA hydrogels disk was put into the 48-well plate in triplicate and incubated at 37 °C for 1 h. L929 cells were seeded into each well at a density of 1 × 10^4^ cells/well and then incubated for 24 h at 37 °C in a 5% CO_2_ atmosphere. The hydrogels were rinsed with sterile physiological saline slightly. The morphology of L929 cells absorbed on the surface of hydrogels was observed from different fields of versions of each well and recorded with an inverted microscope (CKX41, Olympus, Japan). The tissue culture-treated polystyrene (TCPS, Corning) was the control group.

#### 2.3.5 *In vitro* cytocompatibility assay of H-HPMA hydrogel

The cytocompatibility of H-HPMA hydrogels was evaluated using the L929 and HMrSV5 cells by MTT assay. The L929 and HMrSV5 cells were purchased from Bluefcell Biotechnology Development Co., Ltd (Shanghai, China). When L929 or HMrSV5 cells were seeded into a 96-well plate at a density of 1 × 10^4^ cells/well for 24 h at 37 °C in a 5% CO_2_ atmosphere, the culture medium was removed and the fresh complete culture medium containing different concentrations of H-HPMA hydrogel (25, 50, 100, 200, 400 800 and 1600 μg mL^−1^) was added. The cells without treatment served as a control. After incubating for 24 h, each well was replaced with a 200 μL fresh complete medium containing 5 mg/mL MTT and further incubation at 37 °C for 4 h. The supernatant was carefully removed and 150 μL dimethyl sulfoxide (DMSO) was added. The absorbance (A) was measured at a wavelength of 492 nm using a microplate reader (Bio-Rad, Berkeley, CA), and the relative cell viability was determined.

#### 2.3.6 *In vitro* hemocompatibility assay of H-HPMA hydrogel

The hemolysis rate of H-HPMA 1:5 hydrogels was examined using human red blood cell suspension as reported work [33]. In brief, the H-HPMA hydrogel was immersed in normal saline (NS) at 37 °C for 72 h. Then the supernatant was collected to prepare various concentrations of H-HPMA hydrogels extract (10 vol %, 20 vol % and 40 vol %). The equal volume of H-HPMA hydrogel extract was added into 1 mL 2% human red blood cell suspension diluted in NS, following incubating at 37 °C for 3 h. The NS served as the negative control and the distilled water was the positive control group. Subsequently, the suspension was centrifuged at 2000 rpm for 10 min. Its supernatant was collected and photographed. The absorbance at 545 nm was determined. The hemolysis ratio was calculated according to the following equation:

(S1) $$\text{Hemolysis ratio }=\frac{A_{h}-An}{Ap-An}\times 100\%$$

where A_h_, A_p_ and A_n_ were the absorbance values of H-HPMA hydrogels, positive control group and negative control groups, respectively.

### 2.4 Animals

Male Sprague-Dawley (SPD) (220 ± 20 g) rats were at 6–8 weeks old and were purchased from the Laboratory Animal Center of Xi'an Jiaotong University. Animals were fed at an animal facility of IVC class (License no. SCXK (Shaanxi) 2015-002). Free access to food and water, a controllable temperature of 20–22 °C, relative humidity of 50–60% and 12-h day/night cycles were provided. All animal experiments were carried out according to the protocol approved by the Ethical Committee of Xi'an Jiaotong University. And studies were performed in compliance with the Guide for the Care and Use of Laboratory Animals of the Ethical Committee of Xi'an Jiaotong University, Xi'an, China (permit No. XJTU 2019-003).

### 2.5 Prevention of abdominal adhesion *in vivo* by H-HPMA hydrogel

The *in vivo* anti-adhesion evaluation of H-HPMA 2:5 hydrogel was performed using a rat cecum-abdominal adhesion model. The rat model was established in our previous work [34]. In general, the SPD rats were anesthetized with isoflurane inhalation via a small animal gas anesthesia machine (ABS, YUYAN Instruments). After the abdomen hair was shaved, the laparotomy was carried out with a 5 cm long incision along the linea alba. The cecum was gently picked out and abraded constantly with sterile gauze until a petechial hemorrhage appeared on the surface of the cecum. Later, the abdominal wall defect (an area of 1 × 2 cm^2^) was formed by scraping off the peritoneum. Then the cecum injury was attached closely to the corresponding abdominal wall defect by fixing with 3-0 sutures. The control group was washed with 1 mL of physiological saline. For the HA hydrogel and H-HPMA hydrogel group, the wounds were covered with HA hydrogel and H-HPMA hydrogel, respectively. The rats in the normal group were without any treatments. Finally, the muscle and skin were sutured with 4-0 sutures. Rats were subcutaneously injected with penicillin to prevent postoperative infection. All experimental procedures were carried out under sterile conditions. In 5 days and 10 days after surgery, 6 rats for each group were sacrificed to evaluate the anti-adhesion efficiency.

A recurrent adhesion model after adhesiolysis was established as described above. After the establishment of the abdominal adhesion model, the rats were anesthetized on day 5. The abdomen was opened once again and the adhesive sites already formed were detached by the method of a blunt dissection like clinic laparoscopic adhesiolysis. After that, the blood appeared on the separate cecum and abdominal wall. The wounds were formed again. Five days after adhesiolysis, the rats were sacrificed to evaluate the anti-adhesion efficiency.

### 2.6 Gross observation and adhesion score

The rats were euthanized and opened abdomen at three preset time points as described above. The adhesive tissues in the abdominal cavity were observed and recorded. The adhesion score was evaluated in a double-blind manner according to the standard adhesion scoring system [35]. Score 0: no adhesion; score 1: one thin filmy adhesion; score 2: more than one thin adhesion; score 3: thick adhesion with focal point; score 4: thick adhesion with plantar attachment or more than one thick adhesion with focal point; score 5: very thick vascularized adhesion or more than one plantar adhesion.

### 2.7 Histopathological examination

The pathological evaluation of postoperative adhesion between the abdominal wall and cecum was performed using hematoxylin and eosin (H&E) and Masson staining. The corresponding tissues harvested from each group were fixed in 4% paraformaldehyde solution and then were treated with paraffin embedding. Conventional paraffin-embedded tissue sections (5 μm) prepared were stained with Hematoxylin and eosin (H&E) or Masson reagent kit. Besides, the histological evaluation of the major organs (heart, liver, spleen, lung and kidney) on day 5 after the first surgery was also performed with H&E and Masson staining.

### 2.8 Enzyme-linked immunosorbent assay (ELISA)

Whole blood specimens of rats were collected after feeding 5, 10 days and 5 days after adhesiolysis. These specimens were placed at room temperature for 1 h and then were centrifuged at 4000 rpm for 10 min to collect the supernatant. The expression level of tumor necrosis factor-alpha (TNF-α), interleukin 1 beta (IL-1β) and interleukin 6 (IL-6) in the serum of rats were determined with ELISA kits following the protocols.

### 2.9 Quantitative real-time PCR

Quantitative real-time PCR assay was performed with local tissues in the abdominal wall of rats. The rats of each group were sacrificed on day 5, day 10 and day 5 after adhesiolysis, respectively. Then the abdominal wall sample of about 80–100 mg at the adhesion site was collected. Total RNA extraction was carried out by using Trizol reagent. The RNA was reverse transcribed into first‐strand cDNA using a reverse transcription kit according to the manufacture's protocols. Each reaction containing cDNA, 10 μM gene-specific primers, 1 × SYBRTM, and ROXTM was detected with a Real-time fluorescence quantitative PCR instrument (MX3005P, Agilent, USA). The quantitative real-time PCR (q-PCR) primers used for Snai1 were as 5′-CTG GAG AAA CCT GCC AAG TAT G-3′ and 5′-GGT GGA AGA ATG GGA GTT GCT-3'; VEGF-α were 5′-CAA TGA TGA AGC CCT GGA GTG-3′ and 5′-GCT CAT CTC TCC TAT GTG CTG G-3'; TGF-β1 were 5′-GCT GAA CCA AGG AGA CGG AAT A -3′ and 5′-GCA GGT GTT GAG CCC TTT CC -3'; α-SMA were 5′-ACC ATC GGG AAT GAA CGC TT -3′ and 5′-CTG TCA GCA ATG CCT GGG TA -3'; E-cadherin were 5′-ATG AGG TCG GTG CCC GTA TT-3′ and 5′-CGT TGG TCT TGG GGT CTG TGA-3'; Collagen-1 were 5′-CCC AGC GGT GGT TAT GAC TT-3′ and 5′-TCG ATC CAG TAC TCT CCG CT -3'; fibronectin was 5′-AAA CCT CTA CGG GTC GCT G-3′ and 5′-GCG CTG GTG GTG AAG TCA AA -3'; GAPDH were 5′- CTG GAG AAA CCT GCC AAG TAT G -3′ and 5′- GGT GGA AGA ATG GGA GTT GCT -3'. The GAPDH was used as a housekeeping control gene and the relative expressions were calculated using the 2^−ΔΔCT^ method.

### 2.10 Western blotting analysis

The abdominal wall tissue was lysed using Radio immunoprecipitation assay (RIPA) lysis buffer and the lysate was centrifuged and quantitated using the BCA quantification kit following the manufacturer's protocol. A loading buffer was used to adjust the protein concentration. Proteins were separated by 10% sodium dodecyl sulfate-polyacrylamide gel electrophoresis and transferred onto polyvinyl difluoride membranes (Millipore Inc., Billerica, MA, USA). Membranes were blocked with 5% skim milk for 1 h (5% BSA for phosphorylated protein) at room temperature. Next, the membranes were immersed in the specific primary antibodies overnight at 4 °C. Then, membranes were washed four times for 10 min each to remove redundant antibodies, and incubated with HRP-conjugated secondary antibodies for 2 h. Finally, blots were visualized using a chemical imager (MiniChemi 610, Sage Creation Science, China) and an electrochemiluminescence (ECL) kit (B500022, Proteintech Group, USA). The GAPDH was selected to normalize the protein expression. The antibodies and their dilutions are listed in Table S1 (Supporting Information).

### 2.11 Immunofluorescent staining

The immunofluorescent staining was employed to visually observe the expression of mesothelial and mesenchymal cell markers. Briefly, the tissue sections embedded in paraffin (5 μm) were blocked with goat serum to prevent nonspecific antibody binding after deparaffinization and rehydrated, followed by incubation with primary and secondary antibodies. The secondary antibodies were labeled respectively by Alexa Fluor® 594 and Alexa Fluor® 488. Finally, the slides were incubated with 4′,6-diamidino-2-phenylindole (DAPI) staining solution at room temperature for 10min. The images were captured using a fluorescence microscope (NIKON ECLIPSE TI-SR, Japan). The antibodies used were as follows: the primary antibodies included mouse anti-rat t-PA antibody (1:100, cat. no. NBP2-67279; Novus Biologicals, USA), and rabbit anti-rat PAI-1 antibody (1:100, cat. no. NBP1-19773; Novus Biologicals, USA). The secondary antibodies consisted of Alexa Fluor® 594-conjugated goat anti-mouse IgG, (1:100, cat. no. 8890S; CST, USA) and Alexa Fluor® 488-conjugated goat anti-rabbit IgG, (1:100, cat. no. 4412S; Cell Signaling Technology, USA).

In addition, double immunostaining of Occludin and ZO-1, E-cadherin and snail, α-SMA and Cytokeratin 13 were performed following the above procedures to investigate the MMT process. The primary antibodies included mouse anti-rat Occludin antibody (1:750, cat. no. GB111401; Servicebio, China), mouse anti-rat ZO-1 antibody (1:800, cat. no. GB111402; Servicebio, China), mouse anti-rat snail antibody (1:1600, cat. no. GB11260; Servicebio, China) mouse anti-rat α-SMA antibody (1:300, cat. no. GB111364; Servicebio, China), mouse anti-rat Cytokeratin 13 antibody (1:250, cat. no. GB11802; Servicebio, China) and the secondary antibodies were Alexa Fluor® 594-conjugated goat anti-mouse IgG, (1:300, cat. no. 8890S; CST, USA) and Alexa Fluor® 488-conjugated goat anti-rabbit IgG, (1:100, cat. no. 4412S; Cell Signaling Technology, USA).

### 2.12 Biocompatibility and organ toxicity *in vivo* study

The biocompatibility *in vivo* of H-HPMA hydrogel was evaluated by subcutaneous implanting. C57BL/6 mice were injected subcutaneously with hydrogels. Then, the local tissues containing hydrogel were harvested at 1, 2, 3 and 4 weeks after implantation. After that, the subcutaneous tissues that including the hydrogel were subjected to perform with H&E staining and Masson staining and the important organs (heart, liver, spleen, lung and kidney) were also conducted with histological evaluation.

### 2.13 Statistical analysis

All data were presented as means ± standard deviation (SD). One-way analysis of variance (ANOVA) was used to analyze the significance among three or more groups by employing GraphPad Prism version 8.0 software (GraphPad, San Diego, CA). The statistical significance was set as *p* < 0.05. The statistical analysis of adhesion scores without a normal distribution was performed by using Fisher's exact test.

## 3 Results and discussion

### 3.1 Preparation and characterizations of injectable H-HPMA hydrogel

**Fig. 1:**
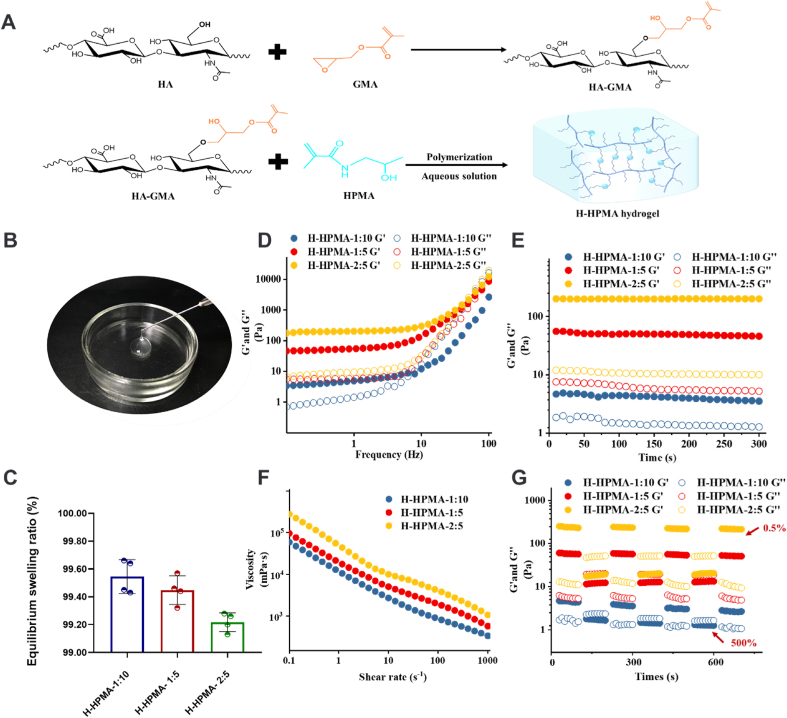
Formation and characterization of H-HPMA hydrogels. (A) The preparation schematic of H-HPMA hydrogels. (B) H-HPMA 1:5 hydrogels passed through sterile syringe needle of 22G, (C) The equilibrium swelling ratios of H-HPMA 1:5 hydrogels. (D) The rheological characterization of H-HPMA hydrogels in frequency-sweep (0.1–100 Hz, strain of 1%), (E) in time-sweep curves (0–5 min, 1 Hz, strain of 1%, 37 °C), (F) Step strain-sweep curves with a low strain at 0.5% and a high strain at 500% (1 Hz, 37 °C). (G) shear rate-sweep (0.1–1000 s^−1^).

As we expected, the injectable H-HPMA hydrogel was fabricated with the methacrylate hyaluronic acid (HA-GMA) and ultra-hydrophilic HPMA by photoinitiation. The HA-GMA served as a long backbone, and numerous HPMA monomers as side chains were conjugated to the main chain of HA as shown in Fig. 1A. During polymerization, a large number of reversible hydrogen bonding networks were established between HA and HPMA. What is more, the ultra-hydrophilic HPMA of the side chain could provide the hydrogel with excellent antifouling ability to resist cell adhesion and nonspecific protein adsorption [18,36]. The HA derivatives HA-GMA was prepared by the ring-opening reaction between the epoxy group of glycidyl methacrylate (GMA) and the alcohol hydroxyl group from HA. The ^1^HNMR spectra of pure HA and HA-GMA were presented in Fig. S1. Compared to the HA, new peaks at 1.82, 5.20, and 5.51 ppm were identified in the spectra of HA-GMA. Moreover, the peak at 1.82 ppm belonged to the methyl proton of the GMA moiety, and two resonance peaks at 5.20, and 5.51 ppm were attributed to the protons of acryloyl double bonds (-C=CH_2_) in the GMA, which demonstrated that the HA-GMA with unsaturated double bond was successfully synthesized. The degree of methacrylation was about 29.71%, which was calculated according to the ratio of the relative peak integration of the vinyl proton signals of GMA to the methyl protons of HA in the NMR spectra [37]. In another way, the FT-IR was also performed to confirm the chemical structure of HA-GMA and H-HPMA. It is noteworthy that a new strong absorption peak at 1726 cm^−1^ was identified in the spectra of HA-GMA, which belonged to the carbonyl group of the GMA moiety as shown in Fig. S2, further showing that the HA was successfully grafted with GMA. Moreover, compared with HA-GMA, an apparent red shift from 3427 to 3398 cm^−1^ in the stretching vibration of O–H was observed in the spectra of the H-HPMA hydrogel, which indicates that new hydrogen interaction has formed between the HA chains and HPMA [[38], [39], [40], [41]]. On the other hand, the characteristic peak of the acryloyl double bond of monomers at 1614 cm^−1^ was clearly visible in the spectra of HA-GMA and HPMA, whereas it disappeared in spectra of the H-HPMA hydrogel, thus confirming the successful polymerization between HA-GMA and HPMA [42].

Clinically, ideal postoperative anti-adhesion products should be injectable and self-healing in order to facilitate surgeon surgery, especially for laparoscopic surgery. Therefore, whether the H-HPMA hydrogel was injectable was determined by performing macroscopic physical extrusion. Briefly, the prepared hydrogel blocks were directly squeezed using a 22G sterile needle several times. We could see that the obtained hydrogel could easily pass through the 22G needle and the extruded parts could quickly fuse seamlessly as shown in Fig. 1B. Simultaneously, the extruded hydrogel was performed with SEM to observe its microstructure to observe its self-fusing property (Fig. S3). Although the initial mechanical property was destroyed by the squeezing, the hydrogel still remained a three-dimensional porous structure. The excellent self-fusing capability of the H-HPMA hydrogel is attributed to abundant reversible non-covalent bond interaction between hydrophilic groups present in HPMA and HA, enabling the hydrogel to quickly restore its original structure. To better understand the hydrophilicity of hydrogel, the equilibrium swelling ratio (ESR) of hydrogel was thoroughly performed in detail. As expected, the ESR of the H-HPMA hydrogels with three different mass ratios were all above 99% (Fig. 1C). The high ESR of the hydrogel was attributed to ultra-hydrophilic HPMA and highly water-retaining HA in structure. Moreover, more HPMA monomer content showed higher ESR, suggesting that the ultra-hydrophilic HPMA plays an important role in the hydrophilicity of the hydrogel.

### 3.2 Rheological and self-fusing capability of H-HPMA hydrogel

The viscoelasticity and self-healing tests of the H-HPMA hydrogel with different mass ratios were performed to further investigate its mechanical properties. In the frequency sweep (Fig. 1D), both storage modulus G′ and loss modulus G″ values for H-HPMA 1: 5 and 2: 5 hydrogels changed negligibly and no intersections were observed in the test frequency range from 0.1 to 10 Hz, indicating that both H-HPMA 1: 5 and 2: 5 hydrogels have a stable gel-like state at a low frequency. For the H-HPMA 1: 10 hydrogel, the G′ and G″ value slightly increased with the increment of frequency, and the G″ > G′ was found when the frequency was over 10 Hz, mirroring the hydrogel network was disturbed and exhibited a sol-like state. On the other hand, the G′ values of the H-HPMA 1: 5 and 2: 5 hydrogels were higher than the G″ values, suggesting that the hydrogels maintain a stable solid‐like (elastic) state. Then, the time-sweep tests were conducted to confirm the stability of the hydrogel. In Fig. 1E, H-HPMA 1: 10, 2: 5, and 1: 5 hydrogel could keep stable G′ and G″ values during the test time (0–300s). The G′ of H-HPMA hydrogel increased from 4 to 200 Pa with the amount of HA-GMA increased. This may have been caused by the increase in the dynamic hydrogen bond density, resulting in a higher crosslinking degree and stronger mechanical properties [43].

Furthermore, the rheological property tests of the H-HPMA hydrogel with different mass ratios were conducted to further verify its stability, injectability, and self-healing properties. The injectability of H-HPMA was determined by the shear rate-sweep test. Fig. 1F shows that the H-HPMA hydrogels exhibit excellent shear-thinning behavior. We could clearly observe that the viscosity of H-HPMA hydrogel constantly decreased by near two orders of magnitude as the shear rate increased from 0.1 to 1,000/s. On a macro level, this shear-thinning behavior is also demonstrated in Movie S1 (Supporting Information), further well confirming the preferable injectability of the hydrogel. After it was prepared as described above by sequentially extruding out from different types of needles (18G, 20G, and 22G), the H-HPMA hydrogel could still be easily squeezed out from a sterile needle and reintegrated into an unbroken and transparent gel. This observation suggests that the hydrogel has excellent self-healing ability and injectability. As reported in our previous work, this kind of hydrogel was named self-fused hydrogel [34].

Supplementary data related to this article can be found at https://doi.org/10.1016/j.bioactmat.2022.02.015.

The following is the supplementary data related to this article:

Multimedia component 2 Multimedia component 2

The self-healing property of hydrogels was determined by performing the step-strain-sweep scanning test. The G′ values of the H-HPMA hydrogel 1: 5 and 2: 5 were simultaneously recovered back to original values when the 500% high strain was switched to the 0.5% low strain, and no obvious changes were observed after three cycles of the test. Similarly, when exposed to the high strain, the G′ values were less than G″ values for the of H-HPMA hydrogel 1: 10 was observed, indicating the network structure of the hydrogel was ruptured and showed a sol-like state. However, when the strain was restored to at the low strain, the initial G′ value of the H-HPMA hydrogel 1: 10 couldn't immediately restore and the G′ value reduced slightly with the increment of cycle times (Fig. 1G). This observation showed that the H-HPMA hydrogel 1: 10 has poor self-healing properties. After the internal structure was damaged, the H-HPMA hydrogel 1: 10 could not be fully restored to the primary gel-like state like the H-HPMA hydrogel 1: 5 and 2: 5.

### 3.3 Anti-fouling capability and *in vitro* cytocompatibility evaluation

**Fig. 2:**
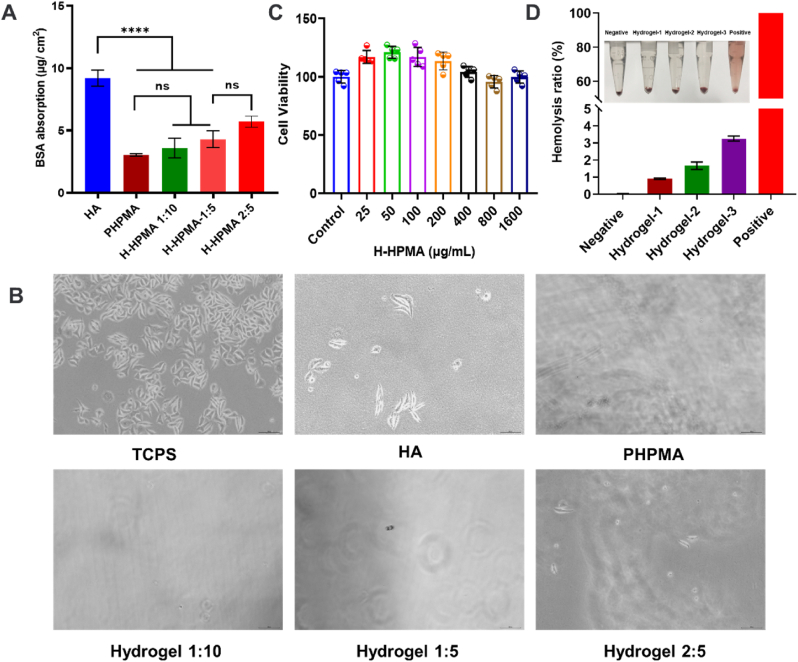
Antifouling ability, cell viability and hemolysis ratio of H-HPMA hydrogels. (A) Protein adsorption of H-HPMA 1:10 hydrogel, H-HPMA 1:5 hydrogel and H-HPMA 2:5 hydrogel. (*p* > 0.05, n = 3). (B) Cellular attachment of L929 on tissue culture polystyrene (TCPS), HA hydrogel, PHPMA hydrogel, H-HPMA 1:10 hydrogel, H-HPMA 1:5 hydrogel, and H-HPMA 2:5 hydrogel (n = 3) (C) Cytotoxicity of H-HPMA 1:5 hydrogel with different concentrations. (n = 5). (D) Hemolysis ratio of different concentrations of H-HPMA 1:5 hydrogel extracts. Normal saline was the negative control, and distilled water was a positive control; Hydrogel-1, Hydrogel-2 and Hydrogel-3 stood for the 10 vol %, 20 vol % and 40 vol % hydrogel extract. All data are presented as mean ± SD (n = 3 per group).

Next, the antifouling ability of the H-HPMA hydrogel was comprehensively evaluated. When peritoneum is damaged or stimulated during surgery, the single layer of mesothelial cells lines would be induced to MMT, acquiring the myofibroblast-like phenotype and accompanying the exudation of extracellular matrix (ECM) components [44,45]. Then the fibrin and fibroblasts accumulate on the wound, causing the permanent adhesion formation [46]. Thus, preventing protein resistance and fibroblast adhesion in the initial stage is essential to effectively avoid postsurgical adhesion. The anti-protein absorption capability and cell attachment resistance of the H-HPMA hydrogel was determined using the bovine serum albumin (BSA) and fibroblast L929 cells. As shown in Fig. 2A, the surface of pure HA hydrogel absorbed 9.21 μg cm^−2^ protein when treated with the BSA solution for 2 h. On the other hand, the absorption amount of the HPMA hydrogel was 3.03 μg cm^−2^, showing the best anti-protein absorption effect. Similarly, the H-HPMA hydrogel also absorbed a rather low amount of BSA. The absorption amount of the H-HPMA hydrogel 1:10, 1:5, and 2:5 were only 3.59 μg cm^−2^, 4.30 μg cm^−2^, and 5.71 μg cm^−2^, which were much lower than those of the HA hydrogel group, indicating that the H-HPMA hydrogel can considerably improve the protein resistance capability of the HA hydrogel. It is mainly attributed to the ultra-hydrophilic polymer HPMA that could form a dense hydration layer on the surface after a strong interaction between the hydrogen bond and water molecules. The surficial water barrier effectively prevented protein absorption. Therefore, we concluded that the introduction of HPMA facilitates the hydration barrier formation, further interfering with the protein adsorption and cell adhesion, and accordingly leading to a satisfactory anti-adhesion effect.

To further illustrate this, the prevention of H-HPMA hydrogel for fibroblast adhesion was evaluated. The tissue culture polystyrene (TCPS) was considered as the positive control group. After incubation with L929 cells for 24 h, the cells on TCPS grew with adherence and proliferation, showing an obvious differentiation tendency (Fig. 2B). On the other hand, spreading of L929 cells to some extent was observed on the HA hydrogel, but almost no cells were observed on the H-HPMA hydrogel and the pure HPMA hydrogel apart from a few L929 cells with an initial spherical shape. This result indicates that the H-HPMA hydrogel could effectively prevent fibroblast cell adhesion. When L929 cells underwent adherent growth, they extruded some charged protein promoting adhesion for the substrate, thus improving the adhesive ability. Nevertheless, the H-HPMA hydrogel contained a large amount of terminal hydroxyl from the HPMA structure, which could easily form strong hydrogen bonds with water molecules and establish a complete hydration barrier. The hydration layer effectively avoided the adhesion of secreted proteins on the surface of the substrate and weakened the adhesion effects of cells, thereby showing a promising resistance performance to nonspecific cell adhesion. These results indicate that the H-HPMA hydrogel effectively prevents postsurgical adhesion by impeding the nonspecific protein absorption and decreasing the interaction between fibroblasts and the hydrogel surface.

In addition, the cytocompatibility of biomaterials was determined for *in vivo* application. After incubation with L929 and human peritoneal mesothelial (HMrSV5) cells for 24 h, the cell compatibility of the H-HPMA hydrogel was evaluated by performing the MTT assay. As shown in Fig. 2C and Fig. S4., both cell types showed over 90% cell viability for different concentrations of the H-HPMA hydrogel solutions, indicating that the H-HPMA hydrogel exhibited favorable biocompatibility. On the other hand, the H-HPMA hydrogel directly contacted the wound when applied as a barrier on the peritoneal trauma, so the hemocompatibility must be considered. Rabbit and human erythrocyte suspensions were selected to evaluate hemocompatibility (Fig. 2D and Fig. S5). After treatment with the H-HPMA hydrogel for 2 h, no obvious hemolysis was observed and all hydrogel extracts showed lower than 5% hemolysis ratios with no obvious significant differences, suggesting that the H-HPMA hydrogel has good blood compatibility, which is safe for postsurgical adhesion *in vivo*.

### 3.4 H-HPMA hydrogel prevented postoperative adhesion *in vivo*

**Fig. 3:**
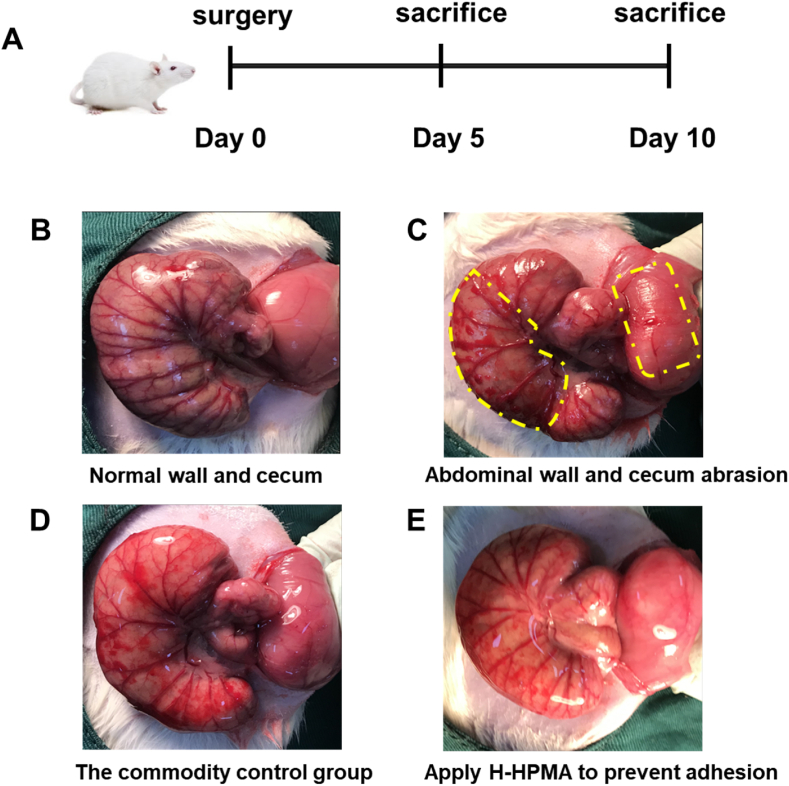
The establishment of a rat sidewall defect–cecum abrasion adhesion model and the application of commercial HA and H-HPMA hydrogel onto the defects. (A) Schematic of the experimental schedule. (B) The normal abdominal sidewall and cecum. (C) The abdominal sidewall and cecum defect model, as yellow rectangles indicate. (D) The commercial HA hydrogel was as the commodity control group and (E) the H-HPMA hydrogel were applied to the injured abdominal wall and cecum.

**Fig. 4:**
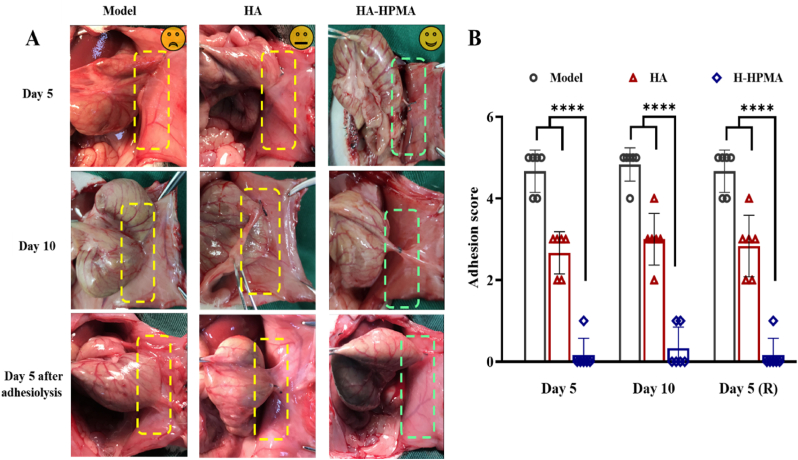
Evaluation of first adhesion prevention on days 5, days 10 and recurrent adhesion prevention on days 5 after adhesiolysis. (A) postoperative adhesions were observed in the control and HA groups while no adhesion was observed in rats treated with H-HPMA on days 5, days 10 and days 5 after adhesiolysis. The yellow rectangle: the adhesion area. The green rectangle: no adhesion area. (B) Adhesion score of the different degrees in each group on the predetermined time points. Day 5 (R) represented for days 5 after adhesiolysis. (n = 6).

Considering the comfortable self-fusing property and excellent anti-fouling capability aforementioned, the H-HPMA hydrogel 1:5 was selected to perform the *in vivo* postoperative anti-adhesion experiment. Furthermore, the preventive efficiency for postoperative adhesion was evaluated by establishing a cecum abrasion-abdominal wall defect model using rats as reported previously [33]. The abdominal cavity was opened on day 0 (Fig. 3A). Then the cecum abrasion was performed by rubbing the cecum until the blood spots appeared on the injury site, and the abdominal wall defect was created by scraping off the surface peritoneum and partial muscle tissue on day 0 (Fig. 3B and C). The administration group consisted of rats receiving the H-HPMA hydrogel (1 mL) and the positive group consisted of rats receiving 1 mL commercial hyaluronic acid (HA) hydrogel. These two hydrogel types were was applied onto the trauma sites of the cecum and abdomen to prevent the postoperative adhesion, respectively, The negative group consisted of rats receiving no treatment for the abraded cecum and defected abdominal wall. (Fig. 3D and E). Generally, after inducing trauma in the abdominal cavity, the mesothelial layer cells in the injury sites were activated to grow during 24 h, and on day 3 the proliferation of the fibroblasts accelerated and angiogenesis was observed on day 5 [47]. Then, the adhesion occurred and granulation tissue formed, indicating fibrosis and conversion to scar tissue [48]. Therefore, the abdominal cavities of rats were checked on day 5, day 10, and day 5 after adhesiolysis and scored accordingly. As shown in Fig. 4A and Fig. 4B, the serious adhesions were observed between parietal and visceral peritoneal tissues in the control group on day 5 (score 4.8, n = 6), and worse and stubborn attachments were developed on day 10, where the surface tissues of the cecum seriously adhered with a large area of peritoneal epidermis, and even the adjacent mesentery, proximal omentum, and adipose tissues were tightly attached. The adhesive sites could not be separated by slight stretching unless peeling off with sharp tools, thus proving the formation of permanent adhesions. Compared to the control group, we could clearly observe that the adhesive area between the cecum and peritoneal epidermis was reduced to some extent, showing the commercial HA gel could only partially alleviate the adhesion with a score of 2.7. Nevertheless, the attachments still contributed to serious complications such as intestinal stenosis, diarrhea, and intestinal mucosal edema, indicating the unsatisfactory anti-adhesion efficiency of commercial HA hydrogel. On the other hand, the average score of H-HPMA hydrogel was 0.2, signifying an ideal prevention effect of about nearly 100% with no adhesion. No attachment was observed in the epidermis of the abdominal wall and cecum. Importantly, the bleeding points and defects resulting from model establishment completely disappeared and had almost recovered.

Except for the first abdominal surgery, likewise, clinic adhesiolysis would lead to novel trauma and bleeding in the abdominal cavity, inducing recurrent adhesion formation. The incidence of recurrent adhesion is still as high as 80% or more after the laparoscopic minimally invasive therapy. In the process of adhesion recurrence, the secondary traumas caused by adhesiolysis surgery for primary adhesive tissue largely contributed to the formation of more firm and complex adhesions [49]. Actually, because of complex surgical procedures, the prevention of recurrent adhesion is a tougher challenge compared to primary adhesion. However, most commercial drugs or materials only highlight the prevention for first postsurgical adhesion but significant anti-adhesion efficacy for repeated injury after adhesiolysis was rarely reported [50]. Hence, a rat model of repeated injury was developed to evaluate the preventive effects of H-HPMA hydrogel on the recurrent adhesion. As shown in Fig. S6, the recurrent adhesion model was developed followed by the successful establishment of the first cecum abrasion-abdominal wall defect model. On day 5 after the complete dissection of the adhesive tissues between the cecum and abdominal sidewall, the corresponding scores were recorded in detail. After more serious traumas and big bleeding points were formed in the cecum and abdominal wall, 1 mL of the H-HPMA or HA hydrogel was evenly injected onto the surface of the damaged cecum and abdominal wall, and the incision was sutured again. On day 5 after adhesiolysis, as exhibited in Fig. 4A and B, the worse and firmed adhesions were clearly visible and the adhesion score was 4.7. Not only adhesion between the cecal wall and peritoneum but numerous surrounding adipose tissues, circumambient mesentery, and colonic bands attached to the cecum or abdominal wall. The adhesion area was obviously expanded over the pristine established wound, tending to induce more serious complications. Similarly, in the HA group, a poor prevention effect against recurrent adhesion was observed, with an average score of 3.0. On the other hand, no adhesion was observed between the cecum and peritoneum, and identically showed complete healing of the wound with satisfactory anti-adhesion efficacy and an average score of 0.2 after the H-HPMA hydrogel was applied. The above results indicate that the H-HPMA hydrogel demonstrates more significant prevention effects on recurrent adhesion than the HA hydrogel.

### 3.5 Histopathological examination

The corresponding cecum and abdominal walls in each group were collected to perform pathological analysis by using hematoxylin and eosin (H&E) and Masson's trichrome (Masson) staining. For the primary adhesion model on day 5, a considerable amount of adhesive tissues were observed in H&E and Masson staining. The smooth muscle of the cecum blended with the peritoneal serous membrane was filled with plenty of connective tissues, causing the disappearance of the boundary between them. The proliferation of fibrous connective tissue is accompanied by newly generated vessels. Granulation tissue hyperplasia, nodular thickening of the peritoneal serous membrane, and infiltration of inflammatory cells were clearly observed in adhesive sites. Furthermore, the Masson staining showed that abundant collagen fiber deposition (blue), collagen hyperplasia, and disordered arrangement had generated in the adhesive sites indicating that permanent adhesions were formed. Similarly, for the rats treated with the HA hydrogel, serious connective tissue hyperplasia still occurred between the damaged serous membrane of the cecum and the injured peritoneal layer of the abdominal wall. It demonstrated a mild fibroproliferative response, connective tissue fibrosis, and collagen deposition in the adhesion sites, suggesting that the HA hydrogel simply exerted a limited therapeutic effect on postoperative adhesions. On the other hand, compared with the control group, the cecum and abdominal walls were completely separated without any adhesion in the H-HPMA hydrogel treatment group. Importantly, either the cecum or abdominal wall did not have an obvious inflammatory response and fibrotic connective tissues, indicating that the bruised cecal serous membrane, the damaged peritoneum, and muscles have recovered. The regenerated integrated mesothelial cell layer that is analogous to the normal tissue was observed on the top of the cecum and peritoneum. The presence of this mesothelial cell layer indicates that when applied to the rat sidewall defect–cecum abrasion adhesion model, the H-HPMA hydrogel can efficiently prevent postoperative adhesion and rapidly heal the peritoneum within 5 days.

**Fig. 5:**
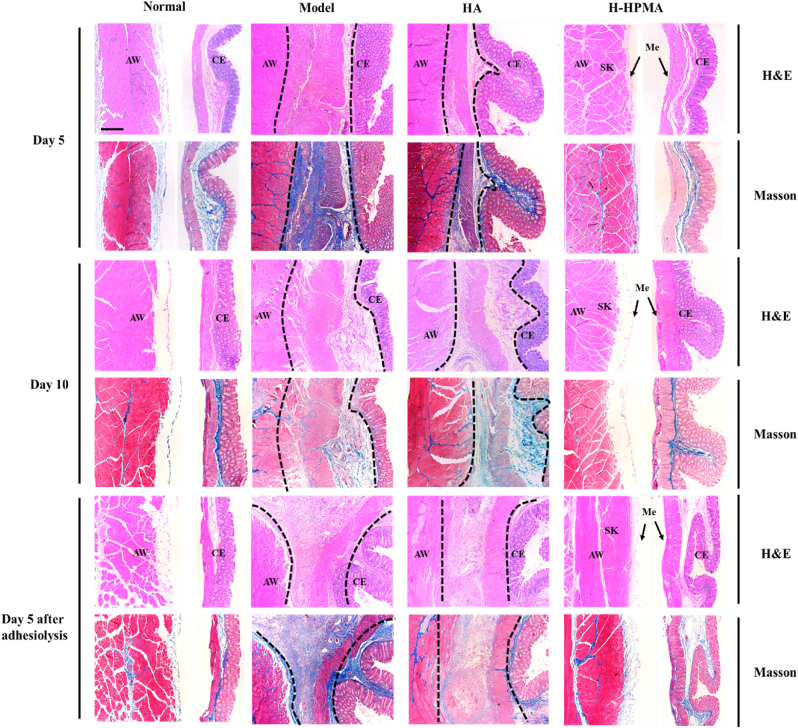
Representative H&E and Masson trichrome staining of adhesive tissues from different groups on day 5, day 10 after first surgery and day 5 after adhesiolysis respectively. AW: abdominal wall; CE: cecal mucosa; Me: mesothelial layer; SK: skeletal muscle of AW. The deposited collagen in the adhesion site was stained in blue while muscle showed red in Masson trichrome staining. (Scale bar, 200 μm).

For the rats that underwent adhesiolysis surgery, the larger area of fibrous connective tissues, accompanied by more neovascularization was found in adhesion sites of the model and the HA hydrogel group. As shown in Fig. 5, the intense thickening of the abdominal wall and corresponding cecal smooth muscle also occurred, where disordered granulation tissue hyperplasia, abundant fibroblasts proliferation, serious inflammatory cells infiltration, and denser collagen deposition (blue in Masson staining) were clearly observed. As shown in Fig. S7, the external longitudinal muscles and internal circular muscles of the smooth muscle layer from the cecum disappeared in the model group, and merged tightly with the abdominal skeletal muscles to form a thick connective tissue with dense collagen deposition, indicating that the adhesiolysis aggravated adhesion formation. Compared with the model group, the longitudinal muscle layer of the cecal smooth muscles in the HA group completely fused with abdominal skeletal muscles, and partial circular muscles could be distinguished. The fibroproliferative response with the submesothelial accumulation of spindle-like cells and fibrosis were developed, indicating that the commercial HA hydrogel could not efficiently prevent recurrent adhesion that resulted from adhesiolysis. On the other hand, in the H-HPMA group, a regenerative mono-layered mesothelium similar to normal tissues appeared in the peritoneum (the black arrow), in which no evident inflammatory cells and deposited collages was observed (Fig. 5). The outer longitudinal muscle and inner circular muscle layer of the cecum tissue were evidently visible (Fig. S8). These results showed that the H-HPMA hydrogel has a satisfactory preventive effect on recurrent adhesion.

### 3.6 Underlying mechanism of H-HPMA hydrogel in preventing adhesions and recurrent adhesions

**Fig. 6:**
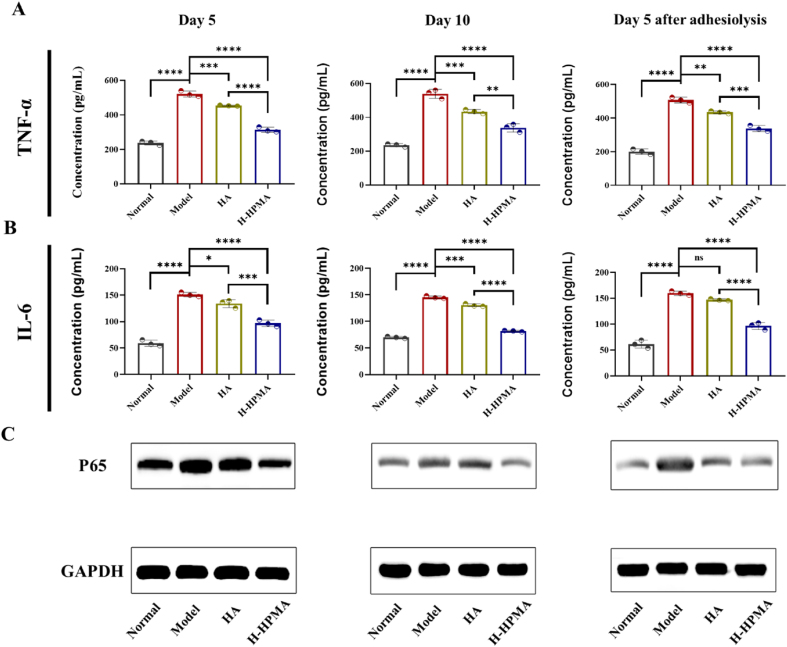
Effects of the H-HPMA hydrogel on nuclear factor κB (NF-κB) signal pathway. A, B) The concentration of TNF-α and IL-6 in serum on day 5,10 postoperation and day 5 after adhesiolysis; C) The expression of p65 protein in the injured tissue on day 5, 10 postoperation and day 5 after adhesiolysis. All data are presented as mean ± SD (n = 4 per group); the ns means no significant difference; *p < 0.05; **p < 0.01; ***p < 0.001; ****p < 0.0001.

The peritoneal inflammatory response induced by the NF-κB pathway plays a dominant role in postoperative peritoneal adhesion [51]. When the wound was formed in the abdominal wall and cecum, damaged peritoneal mesothelial cells produced an inflammation-related immune response, leading to the activation of the NF-κB pathway and the secretion of abundant downstream pro-inflammatory cytokines [52]. The overexpression of inflammatory cytokines such as representative TNF-α, IL-6 and IL-1β would slow wound repair, aggravate connective tissue proliferation and induce inflammation inflation [53]. Furthermore, long-term inflammation is conducive to the mesothelial-mesenchymal transition of peritoneal mesothelial cells that is a key process leading to peritoneal fibrosis [54,55]. It is well known that HA has excellent anti-inflammatory effects and that can promote wound healing [56,57]. Therefore, we expect that H-HPMA hydrogel can also regulate the inflammation response and accelerate peritoneum repair, which could be greatly conductive to prevent peritoneal adhesion. The anti-inflammation effects of H-HPMA hydrogel were determined by investigating the expression of NF-κB and related cytokines. As shown in Fig. 6, compared to the normal group, the p65 expression in the model group significantly increased in the adhesive site, whereas the overexpression of p65 was evidently decreased in the H-HPMA groups, indicating that the H-HPMA could efficiently down-regulate the inflammatory signaling pathway of NF-κB, thereby decreasing relative pro-inflammatory cytokines expression. To further confirm the inhibitory effects of the H-HPMA hydrogel on the inflammatory response, the expression of the corresponding downstream representative cytokine was determined using enzyme-linked immunosorbent assay (ELISA). Compared with the normal group, the TNF-α and IL-6 expression significantly increased in the model group. On the other hand, when the HA or H-HPMA hydrogel was applied on the wound, the TNF-α and IL-6 expression were remarkably reduced, and the H-HPMA hydrogel demonstrated more outstanding inhibitory effects on the inflammatory response induced by the damaged peritoneal mesothelial cells than those in the HA group. Moreover, IL-1β expression also was determined as shown in Fig. S9, which showed a similar tendency. These results suggest that the H-HPMA hydrogel could conspicuously regulate the NF-κB signaling pathway by reducing the p65 expression, further decreasing the relative pro-inflammatory cytokines expression to reduce the inflammatory response of peritoneal mesothelial cells, thus inhibiting fibrosis and the formation of postoperative adhesion.

**Fig. 7:**
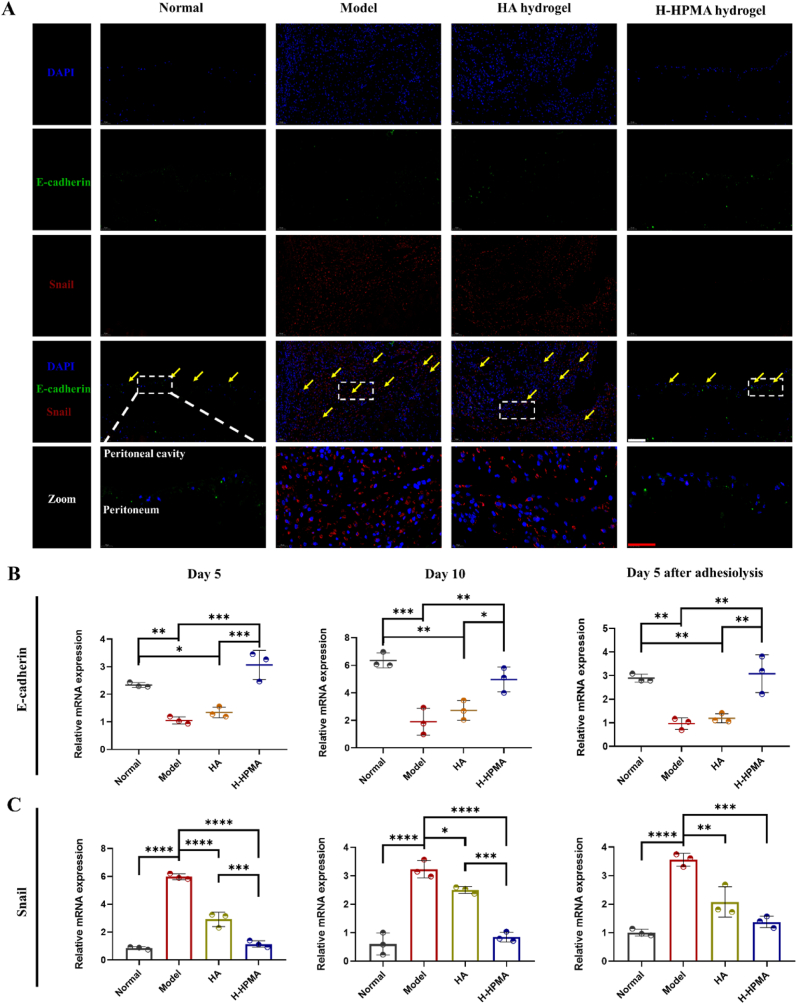
H-HPMA bottlebrush hydrogel ameliorates mesothelial-to-mesenchymal transition in rat abdominal adhesion model. (A) Representative immunofluorescence staining of different groups on day 10 after abdominal surgery. Sections of tissue were stained with DAPI (blue), Snail-Alexa Fluor594 (red) and E-cadherin-Alexa Fluor488 (green). Magnification:200 × ; Scale bars represent 100 μm in the lower magnification images and 50 μm in the higher magnification images. (B) Relative mRNA expression of E-cadherin and, (C) Snail in the adhesive site of different groups on day 5, day 10 after the first surgery and day 5 after adhesiolysis. All data are presented as mean ± SD (n = 3 per group); *p < 0.05; **p < 0.01; ***p < 0.001; ****p < 0.0001.

The peritoneal mesothelial cells (MCs) in the abdominal cavity were are highly associated with abdominal adhesion and tissue fibrosis [58]. When stimulated with a post-surgical wound, the mesothelial-to-mesenchymal transition (MMT) process of mesothelial cells was initiated that led to the accumulation of numerous myofibroblasts in the surgical site, thereby accelerating adhesive tissue formation and further aggravating the fibrosis process [59]. Thus, if the MMT process could be intervened, the postoperative abdominal adhesions and fibrosis might be effectively reduced [60]. It is worth mentioning that in the above animal experiments, the peritoneum completely healed after treatment with H-HPMA hydrogel, and the intact monolayer of regenerative mesothelial cells similar to normal tissue was observed in the cecum and the top of the peritoneum. Therefore, we speculated whether the H-HPMA hydrogel can exert inhibitory effects on MMT. To confirm this speculation, the expressions of important protein markers associated with MMT were was determined using dual-immunofluorescence techniques and qRT-PCR analysis, respectively. Fig. 7A showed that the E-cadherin-positive mesothelial mono-layer was well preserved in the normal and H-HPMA hydrogel groups (shown in green). The expression of Snail protein related to mesenchymal cells (shown in red) could be barely observed in the mesothelial zone after treatment with the H-HPMA hydrogel. On the other hand, no evident mesothelial monolayer was observed in the model and HA groups. The tissues obviously fused together in the adhesive sites and abundant proliferative cells were observed. The strong fluorescent expression of the Snail protein was observed in the adhesive zone, showing that the mesenchymal conversion of mesenchymal cells had occurred and generated plenty of mesenchymal cells. Immunofluorescence staining on day 5 after the first surgery and day 5 was presented in Fig. S10 and Fig. S11, respectively. The clear mesothelial monolayer with low Snail expression shows that the H-HPMA hydrogel efficiently inhibited the MMT process and significantly reduced the postoperative adhesion.

It should be noted that the qRT-PCR analysis of E-cadherin and Snail at different time points showed similar results. As shown in Fig. 7B, when with no treatment, the relative mRNA expression of E-cadherin was the lowest and the Snail expression was the highest, indicating that postoperative trauma significantly inhibited the E-cadherin and induced Snail expression, which further contributed to the MMT process. When treated with the HA hydrogel, no significant difference in E-cadherin expression was observed compared to that in the model group, and the Snail expression was decreased to a certain extent. On the contrary, after treatment with the H-HPMA hydrogel, the E-cadherin expression was significantly preserved and Snail expression was remarkably blocked, indicating that the H-HPMA hydrogel has excellent inhibitory effects on the MMT process.

**Fig. 8:**
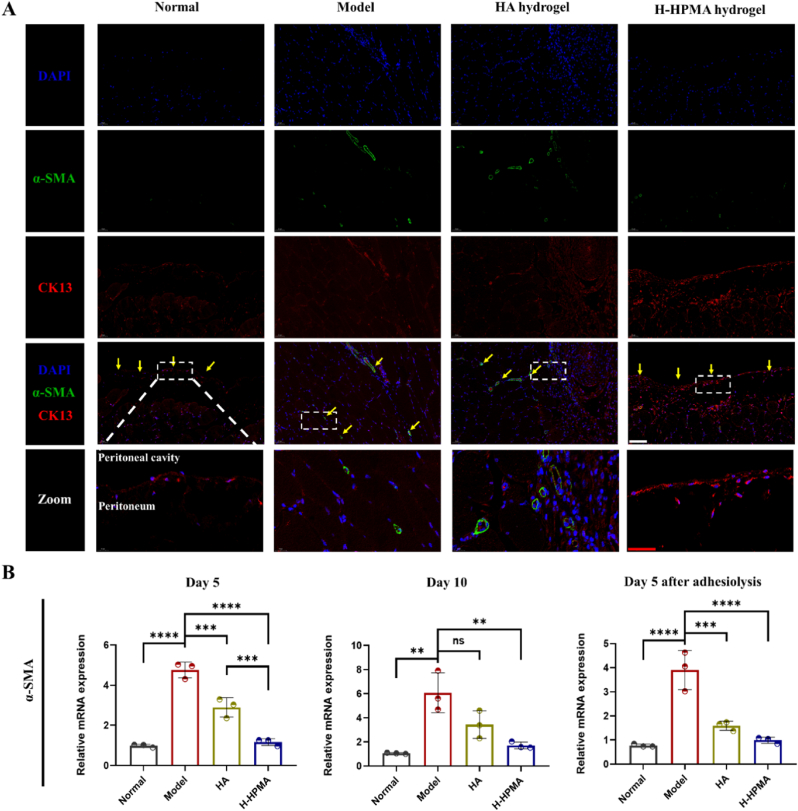
H-HPMA bottlebrush hydrogel ameliorates mesothelial-to-mesenchymal transition in rat abdominal adhesion model. (A) Representative immunofluorescence staining of different groups on day 10 after abdominal surgery. Sections of tissue were stained with DAPI (blue), CK13-Alexa Fluor 594 (red) and α-SMA-Alexa Fluor488 (green). Magnification:200 × ; Scale bars represent 100 μm in the lower magnification images and 50 μm in the higher magnification images. (B) Relative mRNA expression of α-SMA in the adhesive site of different groups on day 5, day 10 after first surgery and day 5 after adhesiolysis. All data are presented as mean ± SD (n = 3 per group); *p < 0.05; **p < 0.01; ***p < 0.001; ****p < 0.0001.

Additionally, the cytokeratin and α-SMA are also important mesothelial and mesenchymal markers in the process of MMT. In terrible peritoneal adhesive sites accompanying binding between the peritoneum and visceral layers, the cytokeratin expression tended to be significantly downregulated and the α-SMA expression appeared to be upregulated, demonstrating that the MMT had undergone. To confirm the inhibitory effect of the H-HPMA hydrogel on the MMT, the double-immunofluorescence analysis of cytokeratin and α-SMA was performed (Fig. 8A, Fig. S12 and Fig. S13). With the MMT process occurring, the fluorescence of cytokeratin was strongly reduced but that of the α-SMA was obvious in the adhesive site in the model and HA group. On the other hand, when treated with H-HPMA hydrogel, spindle-like mesothelial cells located in the peritoneal sub-mesothelium showed a strong expression of cytokeratin but no evident α-SMA expression. Similarly, in the normal group, the submesothelial cells showed a significant mesothelial-positive expression of cytokeratin but no mesenchymal-positive expression of α-SMA. Furthermore, the relative mRNA expression of α-SMA was quantitatively determined by qPCR (Fig. 8B). Similarly, the mRNA expression of α-SMA significantly decreased in the H-HPMA hydrogel group, suggesting that adhesion can be considerably prevented by inhibiting the MMT process.

**Fig. 9:**
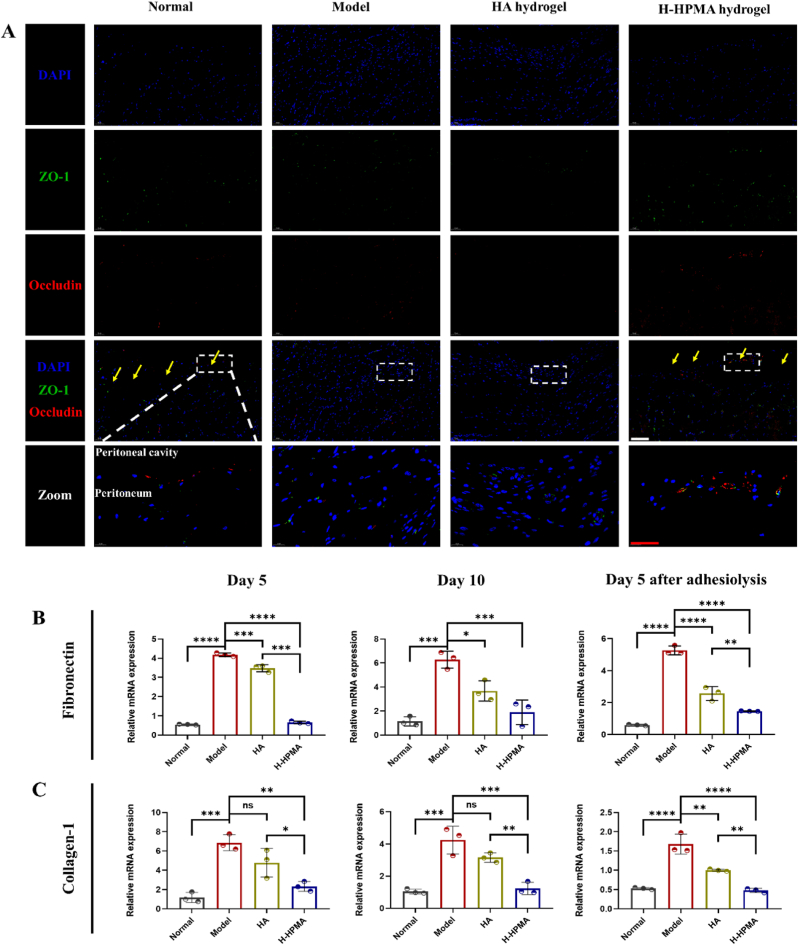
H-HPMA bottlebrush hydrogel ameliorates mesothelial-to-mesenchymal transition in rat abdominal adhesion model (A) Images of immunofluorescence staining on day 10 after adhesiolysis. Sections of tissue were stained with DAPI (blue), occludin-Alexa Fluor® 594 (red) and ZO-1-Alexa Fluor®488 (green). Magnification: 200 × ; Scale bars represent 100 μm in the lower magnification images and 50 μm in the higher magnification images. (B-C) Relative mRNA expression of the fibronectin and collagen-Ⅰ in the adhesive site of different groups on day 5, day 10 after first surgery and day 5 after adhesiolysis. All data are presented as mean ± SD (n = 3 per group); the ns means no significant difference; *p < 0.05; **p < 0.01; ***p < 0.001; ****p < 0.0001.

Moreover, the important MMT markers conductive to MMT such as tight proteins ZO-1 and occludin were also significantly upregulated accordingly (Fig. 9A, Fig. S14 and Fig. S15). In the model and HA groups, no obvious fluorescent expression was observed. On the contrary, significant fluorescence co-located in the peritoneum for the H-HPMA group and the monolayer mesothelial cells was clearly visible, suggesting that complete recovery of the peritoneum in the H-HPMA group. In addition, the relative mRNA expressions of representative markers such as fibronectin, collagen-I, and VEGF-α were also quantitively determined using qPCR (Fig. 9B–C and Fig. S16). We observed that the expression of fibronectin and collagen-Ⅰ significantly increased when the adhesion formed. Interestingly, after applying the H-HPMA hydrogel to the injury site, the expression of fibronectin and collagen-Ⅰ nearly decreased to the normal level, indicating that the peritoneal cells still maintained the mesothelial cell morphology. It further verified that the H-HPMA hydrogel had an effective inhibitory effect on the MMT process that fundamentally reduced the production of myofibroblast-like cells, so as to prevent postoperative abdominal adhesion.

**Fig. 10:**
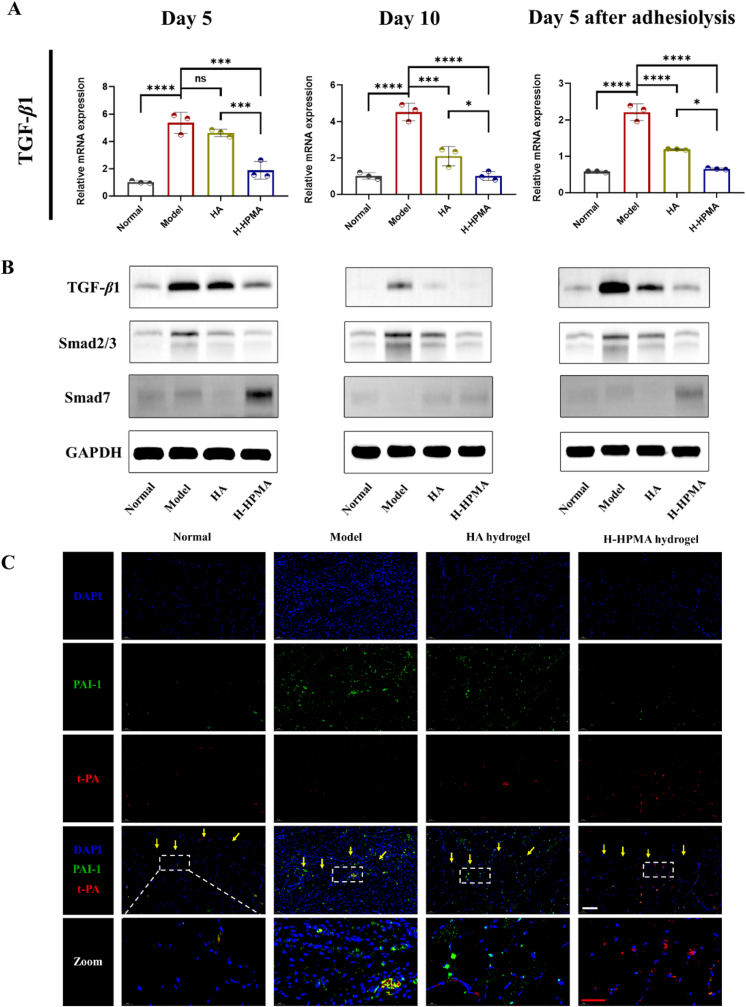
Potential effect on MMT of the H-HPMA hydrogel by regulating the TGF-β1/Smad signal pathway. (A) Relative TGF-β1 mRNA expression in the peritoneal tissues; (B) the expression of TGF-β1, Smad2/3 and Smad7 proteins in relative to the GAPDH in the peritoneal tissue analyzed with western blotting. e,f) Representative immunofluorescence images in the rat peritoneal membrane on day 10 post-surgery. Sections were stained with DAPI (blue), PAI-1-Fluor 594 (red) and t-PA-Alexa Fluor488 (green). Magnification: 200 × ; Scale bars: 100 μm. All data are presented as mean ± SD (n = 3 per group); the ns means no significant difference; *p < 0.05; **p < 0.01; ***p < 0.001; ****p < 0.0001.

Transforming growth factor-β1 (TGF-β1) is the key factor for promoting postoperative adhesion formation and it is also considered the master molecular mediating MMT [61]. Some studies have reported that inhibiting the expression of TGF-β1 can efficiently regulate the pathogenic peritoneal adhesion and fibrosis induced by MMT [62]. Compared to the high expression in the model group in Fig. 10A, the H-HPMA hydrogel significantly alleviated the TGF-β1 mRNA expression almost back to the normal level. These results indicated that the H-HPMA hydrogel had a potential regulatory effect on the MMT process. When the mesothelial cells underwent the MMT process, accompanied by the high expression of TGF-β1, which further activated the downstream Smad2/3, producing a large amount of pro-fibrotic cytokines like plasminogen activator inhibitor-1 (PAI-1), thereby aggravating the MMT process [63,64]. On the other hand, Smad7 is competitively bound to TGF-β1 RI or induced its degradation, avoiding the activation of Smad3, thus inhibiting the TGF-β1 signal pathway and attenuating the MMT process [65]. Therefore, the potential effects of H-HPMA hydrogel on the TGF-β1 signal pathway were confirmed by western blotting. As shown in Fig. 10B, we observed that the TGF-β1 and Smad2/3 had a significant and high expression in the model group. In the HA group, their expression was slightly reduced. On the contrary, the H-HPMA hydrogel showed a markedly lower expression compared with the rats without treatment, and no significant difference was found between the H-HPMA hydrogel and the normal group. Notably, compared to HA hydrogel, the Smad7 expression of the H-HPMA hydrogel group was also significantly increased, indicating that the H-HPMA hydrogel can efficiently decrease the Smad2/3 expression contributing to MMT, and significantly increase the Smad7 expression, which further blocks the MMT process.

Plasminogen activator inhibitor-1(PAI-1) and tissue plasminogen activator (t-PA) are also key marker proteins in peritoneal mesothelial cells and mesenchymal cells, which are regulated by TGF-β1 and can participate in the MMT [66,67]. Previous studies reported that TGF-β1 could decrease t-PA and increase PAI-1 production in human mesothelial cells during peritoneal tissue repair [68]. To confirm whether the H-HPMA hydrogel can inhibit the downstream PAI-1 expression of the TGF-β1 signal pathway to regulate the MMT, the double immunofluorescence staining of the peritoneum was performed simultaneously (Fig. 10B, Fig. S17, and Fig. S18). For the peritoneum in the adhesive tissue in the model and HA group, a large amount of PAI-1 expression was apparent, while the t-PA expression was almost negligible in the field. Contrarily, obvious t-PA positive cells but few PAI-1-expressing cells could be observed in that of the H-HPMA hydrogel group, which were similar to the normal group. Hence, based on these results, we concluded the H-HPMA hydrogel could regulate the balance between downstream PAI-1 and t-PA expression of the TGF-β1 signal pathway, further positively intervening MMT to effectively prevent adhesion formation in abdominal surgery.

### 3.7 *In vivo* biocompatibility of H-HPMA hydrogel

**Fig. 11:**
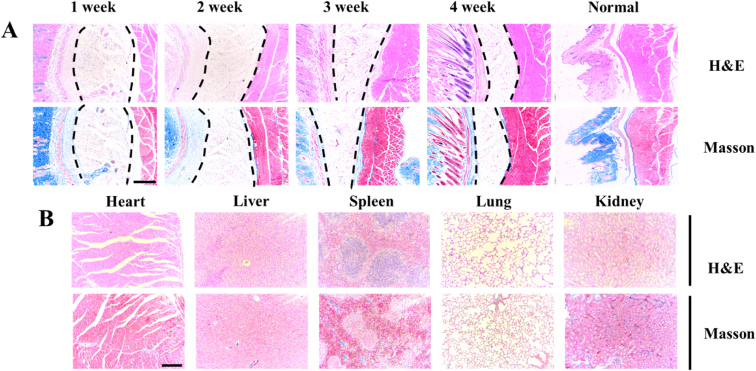
The degradation time and biocompatibility evaluation of H-HPMA hydrogel *in vivo* after subcutaneous implantation. (A) H&E staining and Masson's trichrome staining of the local tissues containing hydrogel at predetermined time points. The zone marked with black dashed lines was the hydrogel area. (B) Histological evaluation of major organs (heart, liver, spleen, lung, kidney) after subcutaneously implanting for 1 week. Magnification: 40 × ; Scale bars: 200 μm.

A high level of biocompatibility for an ideal hydrogel is also necessary when it is used for preventing adhesion. Generally, the implanted hydrogel would result in a severe foreign body reaction. The serious inflammatory infiltration and dense collagen deposits would be observed in the local tissues surrounding the hydrogel. Two to three weeks after implantation, the hydrogel would be encapsulated by the adjacent tissues and formed a dense collagenous capsule [69,70]. The foreign body reaction *in vivo* for H-HPMA hydrogel was performed by using subcutaneous implantation in C57BL/6 mice. By one to two weeks after implantation, only a few inflammatory responses and loose collagen fibers distribution that was stained in blue were observed in the tissues adjacent to the hydrogel (Fig. 11A). When implanted for 3–4 weeks, the hydrogel volume decreased obviously, and the inflammatory cells gradually disappeared. No collagenous capsule was formed at the hydrogel–tissue interface, indicating that the H-HPMA hydrogel exhibited good biocompatibility. This phenomenon could be attributed to its excellent ability of anti-protein absorption by which it can escape from the macrophage phagocytosis. Moreover, from one week to four weeks after subcutaneous injection, the size of the hydrogel gradually decreased over time, suggesting that the H-HPMA hydrogel can be constantly absorbed by the body until complete degradation. After implantation for three to four weeks, plenty of regenerative vessels and hair follicles were clearly observed in the interface, revealing that the H-HPMA might be conducive to revascularization. It could be due to the loosened collagen structure adjacent to the H-HPMA hydrogel, which can further improve the oxygen, nutrient substance metabolism between tissues. The organ toxicity of the H-HPMA hydrogel was also performed using H&E and Masson staining. The major organs of C57BL/6 mice were collected 1 week after subcutaneous injection. As shown in Fig. 11B and Fig. S19, no significant difference was observed between the normal and the H-HPMA group. The neat arrangement of cardiac cells presented complete morphology, and no inflammatory responses were elicited. Similarly, the hepatic sinusoids had no pathological abnormalities. The normal white and red pulp could be seen in the spleen tissue. Besides, in the lung tissue, the expansion or collapse of alveoli and hyperplasia or edema inflammatory cells were not found. The kidney tissue showed healthy renal tubules and glomeruli similar to the normal kidney tissue. These results indicate that the H-HPMA hydrogel has excellent *in vivo* biocompatibility for the tissues and organs that can be an ideal biomaterial for post-surgical adhesion prevention.

## 4 Conclusions

To summarize, a novel injectable H-HPMA hydrogel inspired by bottlebrush was designed and developed by the free-radical polymerization in aqueous solution between the methacrylated hyaluronic acid (HA-GMA) and N-(2-hydroxypropyl) methacrylamide (HPMA) monomer without any chemical crosslinkers. A large number of reversible hydrogen bonding networks between HA and HPMA conferred the H-HPMA hydrogel with excellent self-fused properties and suitable abdominal metabolism time. Furthermore, the introduction of abundant ultra-hydrophilic HPMA provided H-HPMA hydrogel with a higher antifouling capability. It is worth mentioning that the H-HPMA hydrogel established a dense hydrated layer that could rapidly prevent and heal the rat's post-surgical peritoneal adhesions and recurrent adhesions after adhesiolysis within 5 days in the rat model, thus considerably shortening the repair time. Subcutaneous implantation *in vivo* demonstrated its excellent biocompatibility. Importantly, an underlying mechanism preventing peritoneal adhesions by regulating the mesothelial-to-mesenchymal transition (MMT) process was demonstrated. Thus, in this study, we demonstrated that our novel self-fused, antifouling, and injectable H-HPMA hydrogel can be considered for a new approach to developing a peritoneal adhesion prevention barrier.

## CRediT authorship contribution statement

**Jushan Gao:** Conceptualization, Methodology, Investigation, Formal analysis. **Jinpeng Wen:** Investigation, Resources. **Datao Hu:** Investigation, Resources. **Kailai Liu:** Investigation. **Yuchen Zhang:** Resources. **Xinxin Zhao:** Investigation, Resources. **Ke Wang:** Conceptualization, Supervision, Project administration, Funding acquisition.

## Declaration of competing interest

The authors declare no conflict of interest.
