# Supplementary material for: Bottlebrush inspired injectable hydrogel for rapid prevention of postoperative and recurrent adhesion
Source: Bioact Mater. 2022 Feb 21;16:27–46. doi: 10.1016/j.bioactmat.2022.02.015 (PMC8958549; doi:10.1016/j.bioactmat.2022.02.015)
Supplement: Multimedia component 1 [file mmc1.docx]

Supplementary information

**Bottlebrush Inspired Injectable Hydrogel for Rapid Prevention of Postoperative and Recurrent Adhesion**

Jushan Gao, Jinpeng Wen, Datao Hu, Kailai Liu, Yuchen Zhang, Xinxin Zhao, Ke Wang*

School of Pharmacy, Health Science Center, Xi’an Jiaotong University, Xi’an 710061, China.

E-mail:perpetual1003@mail.xjtu.edu.cn

**Supporting Figures**

**
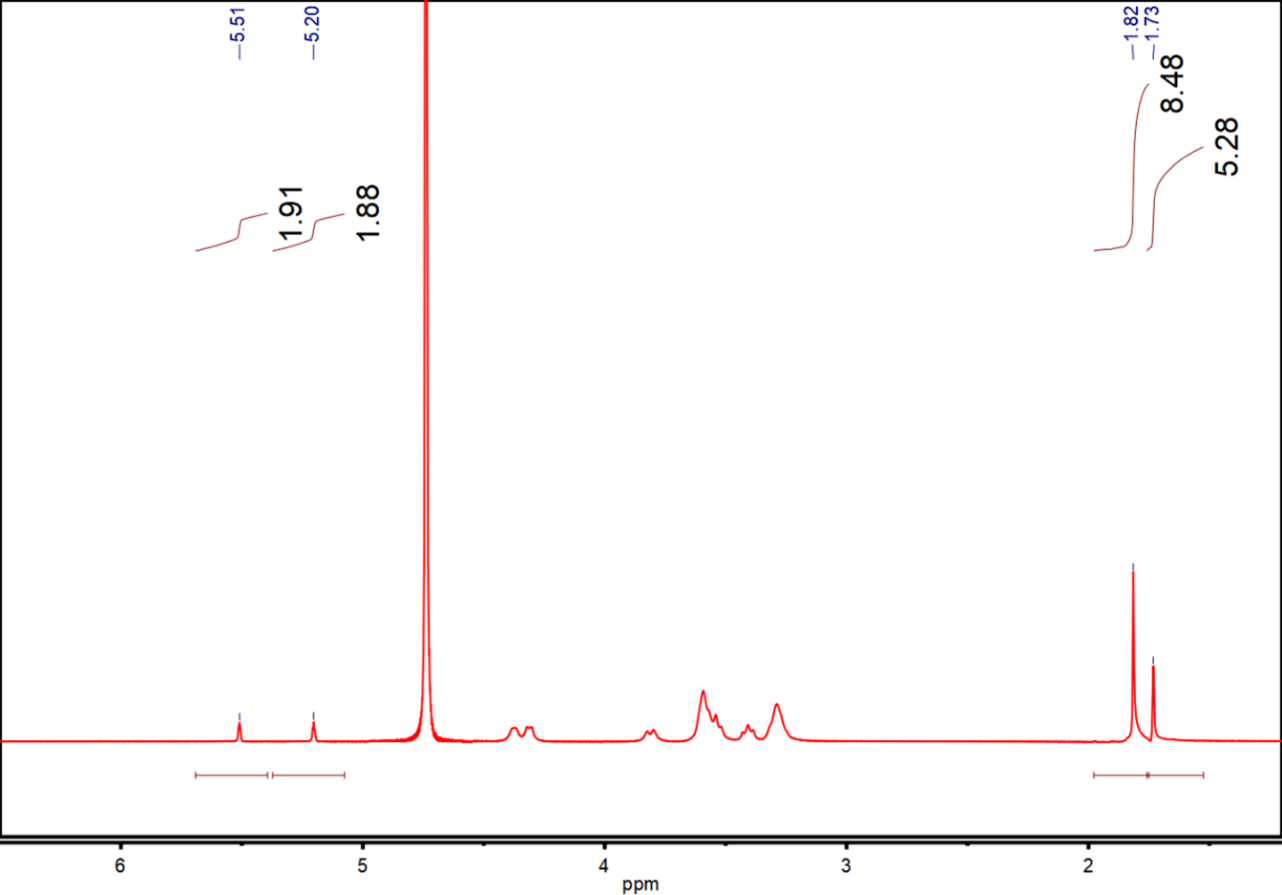
**

**Figure S1.** ^1^H NMR spectra of HA-GMA.

**
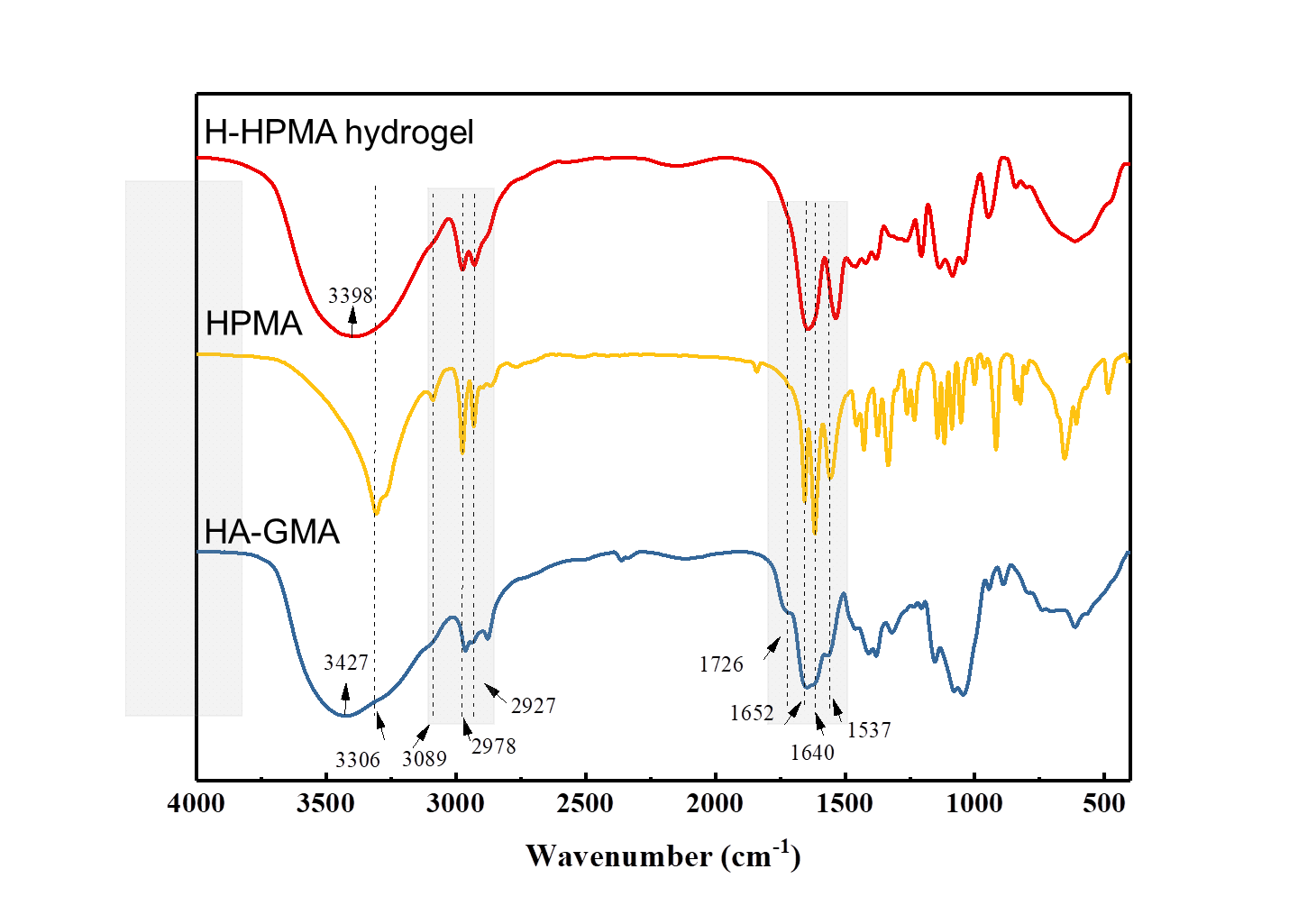
**

**Figure S2.** FT-IR spectra of HA-GMA, HPMA and H-HPMA hydrogel

**
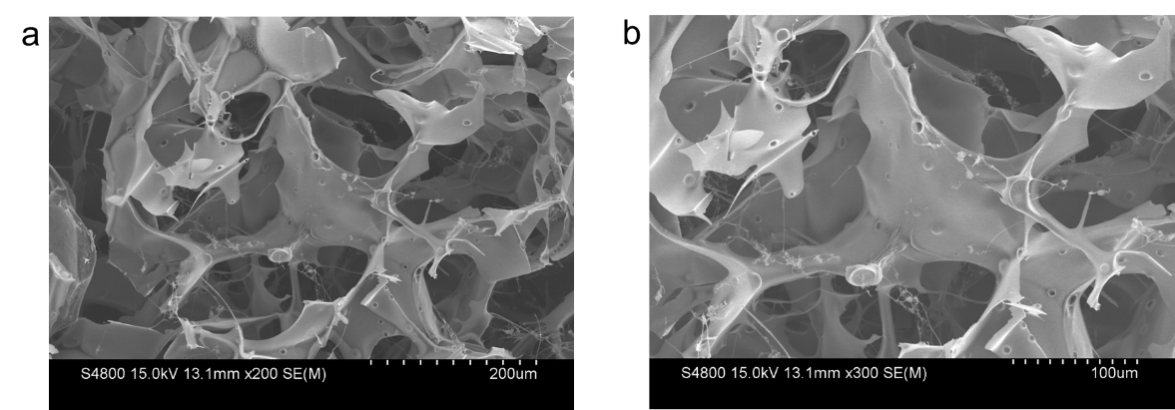
**

**Figure S3.** The SEM images of H-HPMA hydrogel in two different magnifications of (a) 200× and (b) 100×.

**Figure S4.** The HMrSV5 cell viability was treated with the culture media with different concentrations of H-HPMA 1:5 hydrogel for 24 h (n = 4).


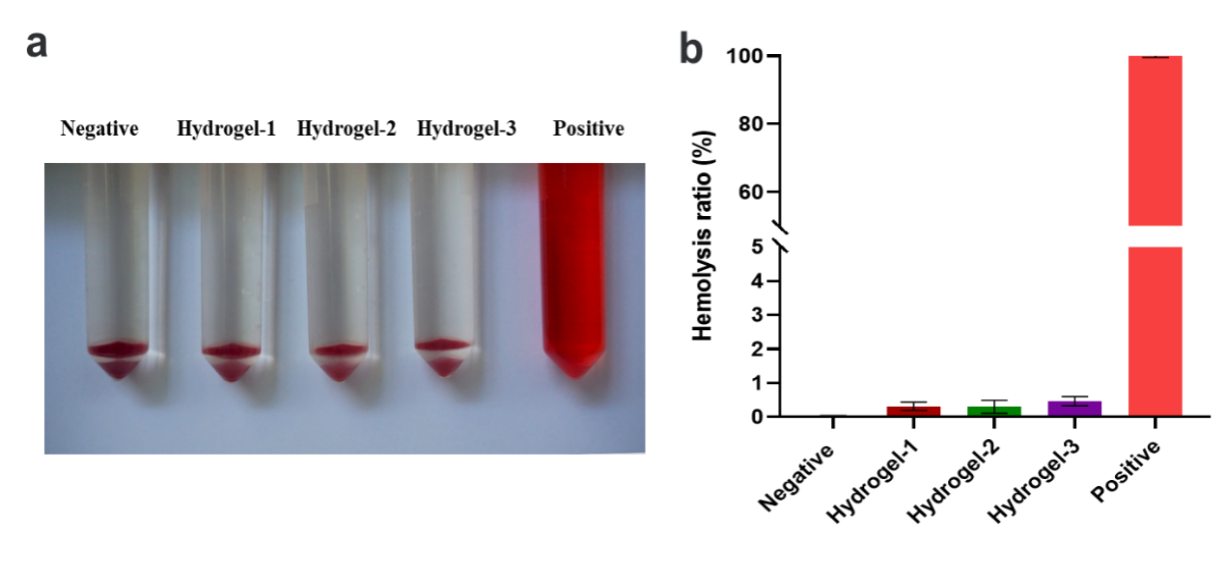


**Figure S5**. Hemolysis ratio of H-HPMA 1:5 hydrogel when performed using rabbit erythrocyte suspension *in vitro* (a) Representative photographs of hemolysis of H-HPMA hydrogel. (b) Hemolysis ratio of H-HPMA hydrogel. Normal saline was the negative control, and distilled water was a positive control; Hydrogel-1, Hydrogel-2 and Hydrogel-3 stood for the 10 vol %, 20 vol % and 40 vol % hydrogel extract. All data are presented as mean ± SD (n=3 per group).


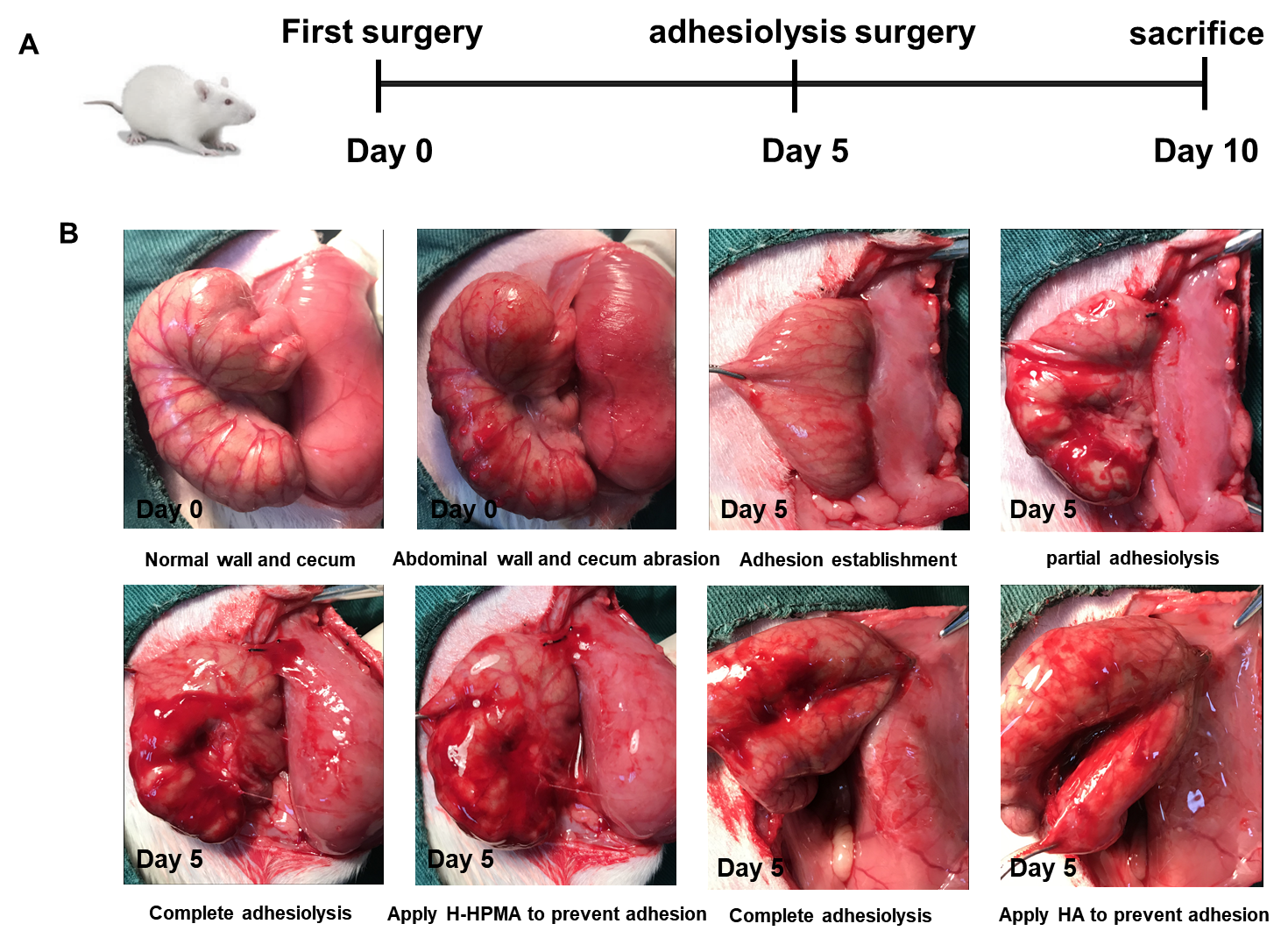


**Fig. S6.** Establishment of a rat recurrent adhesion model to evaluate the antiadhesion efficacy of HA and H-HPMA hydrogel. (A) Schematic of the experimental schedule. (B) Procedures of establishing the rat recurrent adhesion model and applying antiadhesion materials onto the reinjured sites. Abdominal wall defect–cecum abrasion model was established in SD rats at the first surgery (day 0). The established adhesion was lysed at the second surgery and the repeated injury was performed by detaching the abdominal wall and cecum (day 0). Then, H-HPMA and HA hydrogel were applied to the reinjured abdominal wall and cecum.


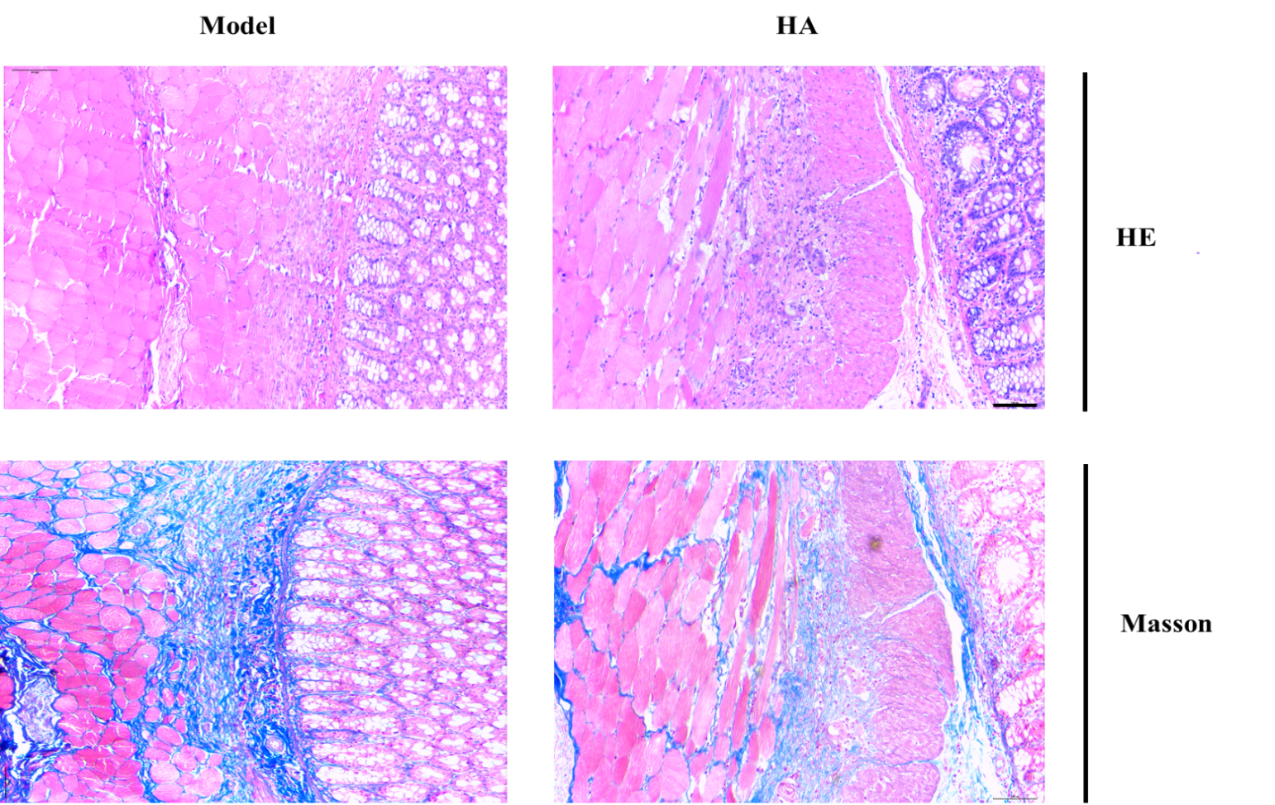


**Figure S7.** H&E and Masson trichrome staining images of adhesive tissue from the model group without treatment and HA group on day 5 after recurrent injury of adhesiolysis for a rat sidewall defect-cecum abrasion model. Scale bar, 100 μm.


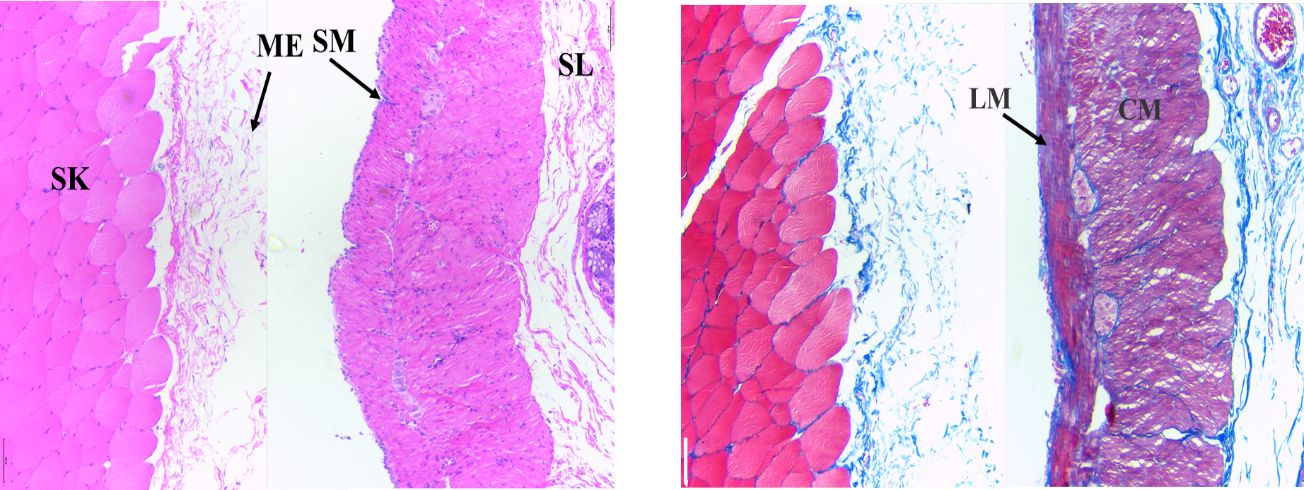


**Figure S8.** Histology images of the abdominal wall and cecum of H-HPMA. Me: mesothelial layer; SK: skeletal muscle; SL: Submucosa layer; SM: visceral smooth muscle; LM: Longitudinal muscle; CM: Circular muscle. Scale bar, 100 μm.


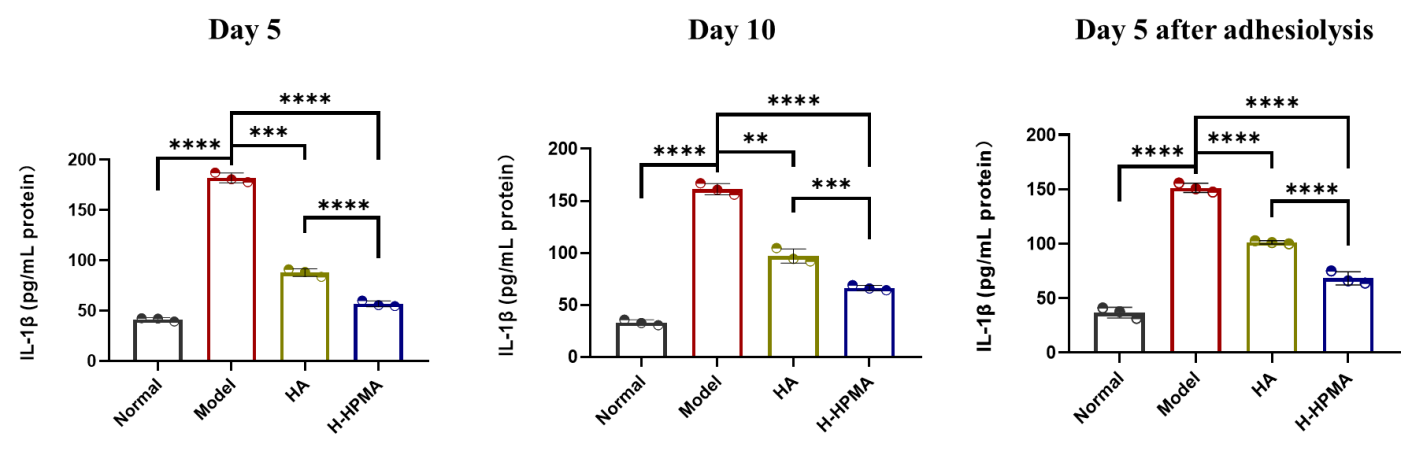


**Figure S9.** The concentration of IL-1β in serum on day 5, 10 postoperation and day 5 after adhesiolysis All data are presented as mean ± SD (n=4 per group); the ns meansno significant difference; **p* < 0.05; ***p* < 0.01; ****p* < 0.001; *****p* < 0.0001.


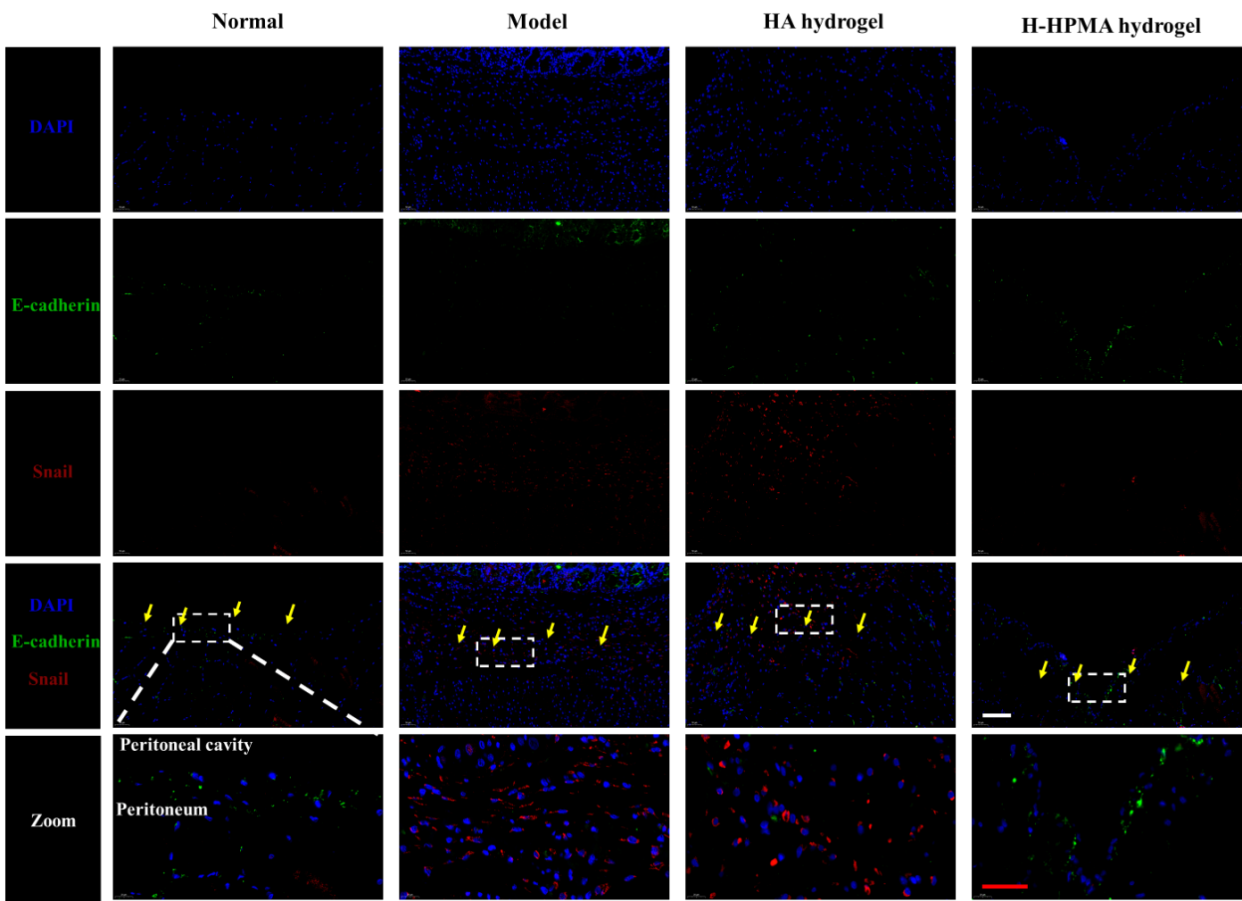


**Figure S10.** Representative immunofluorescence staining of different groups on day 5 after the first surgery. Sections of tissue were stained with DAPI (blue), Snail-Alexa Fluor®594 (red) and E-cadherin-Alexa Fluor®488 (green). Magnification:200×; Scale bars represent 100 μm in the lower magnification images and 50 μm in the higher magnification images.


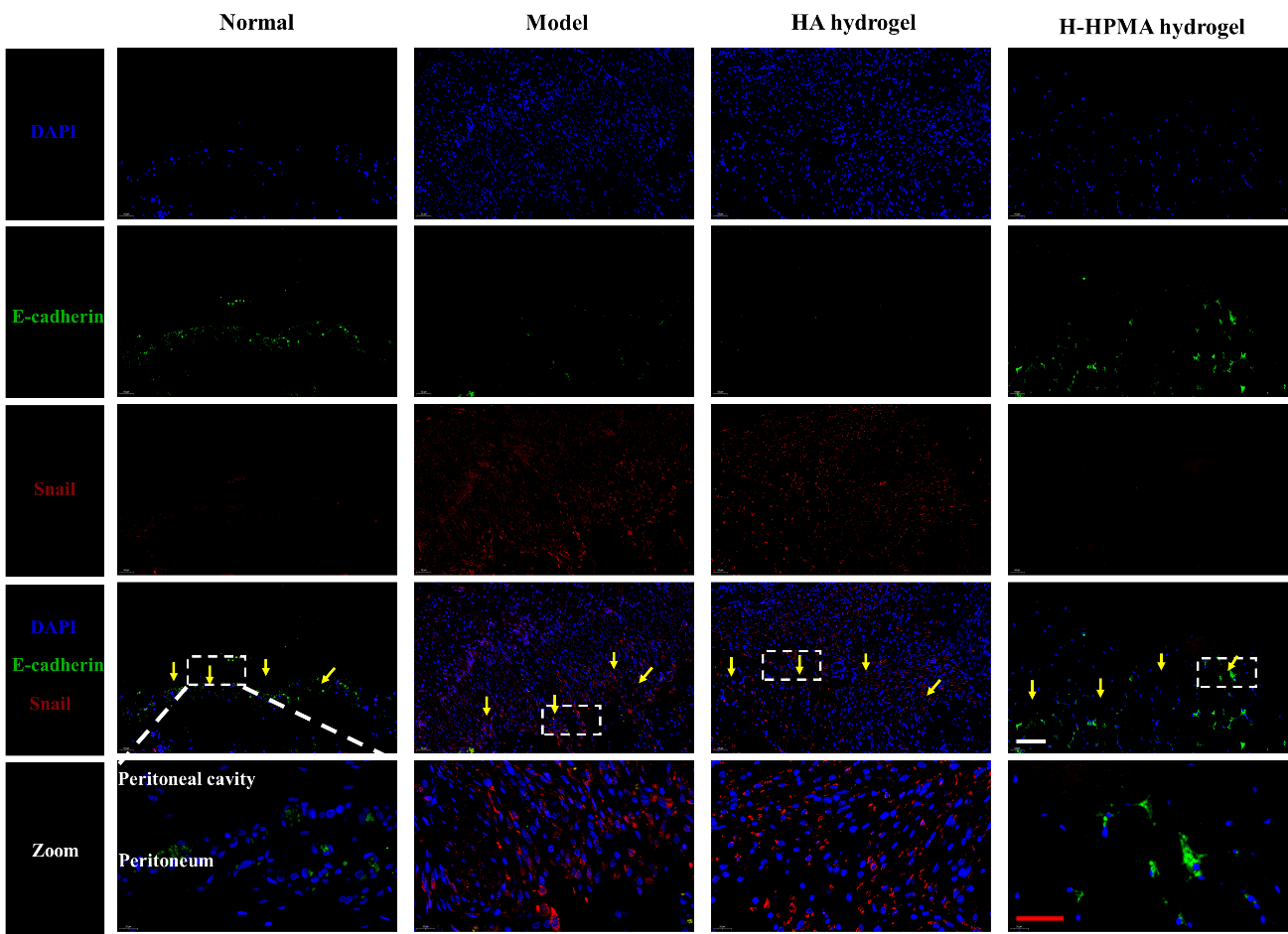


**Figure S11.** Representative immunofluorescence staining of different groups on day 5 after adhesiolysis. Sections of tissue were stained with DAPI (blue), Snail-Alexa Fluor®594 (red) and E-cadherin-Alexa Fluor®488 (green). Magnification:200×; Scale bars represent 100 μm in the lower magnification images and 50 μm in the higher magnification images.


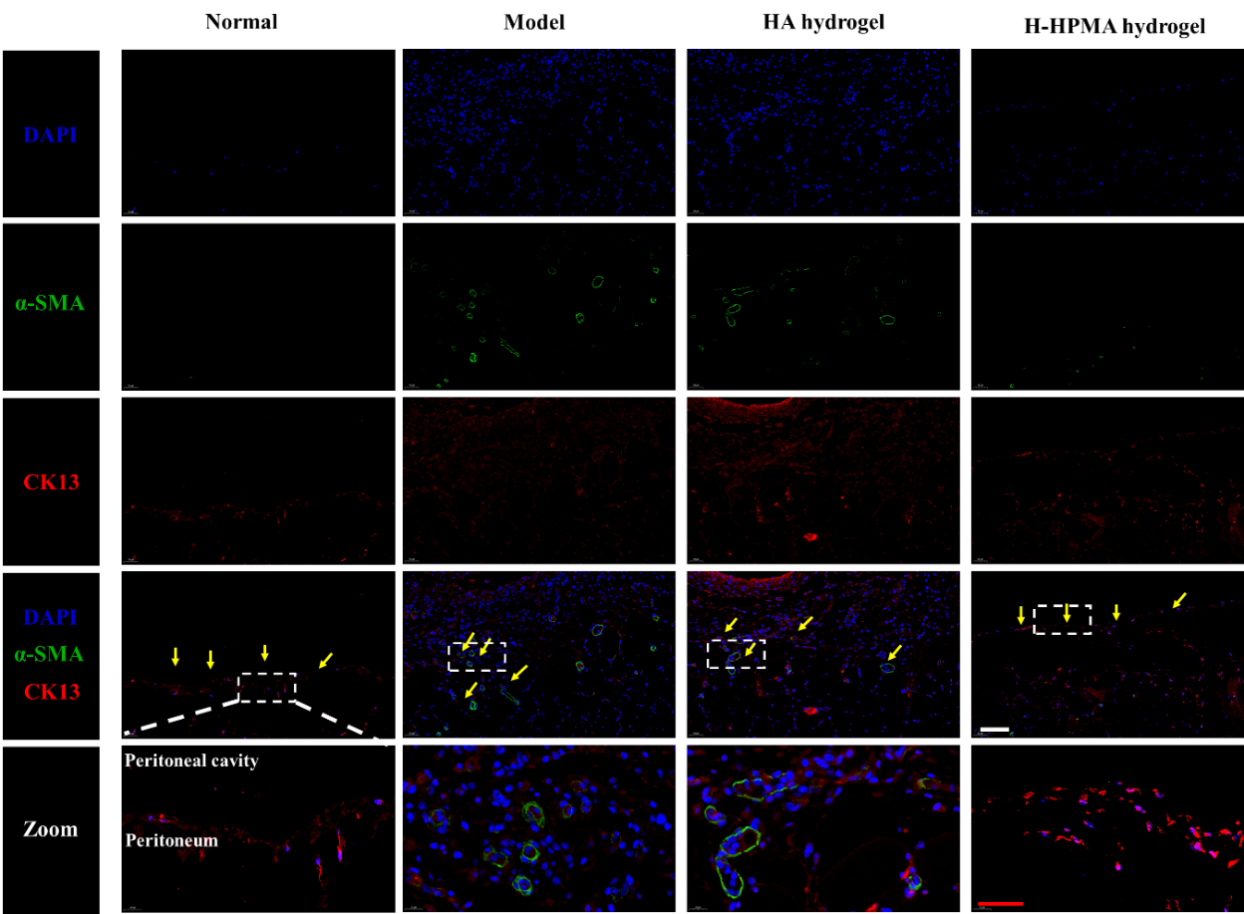


**Figure S12.** Representative immunofluorescence staining of different groups on day 5 after first surgery. Sections of tissue were stained with DAPI (blue), CK13-Alexa Fluor®594 (red) and α-SMA-Alexa Fluor®488 (green). Magnification:200×; Scale bars represent 100 μm in the lower magnification images and 50 μm in the higher magnification images.


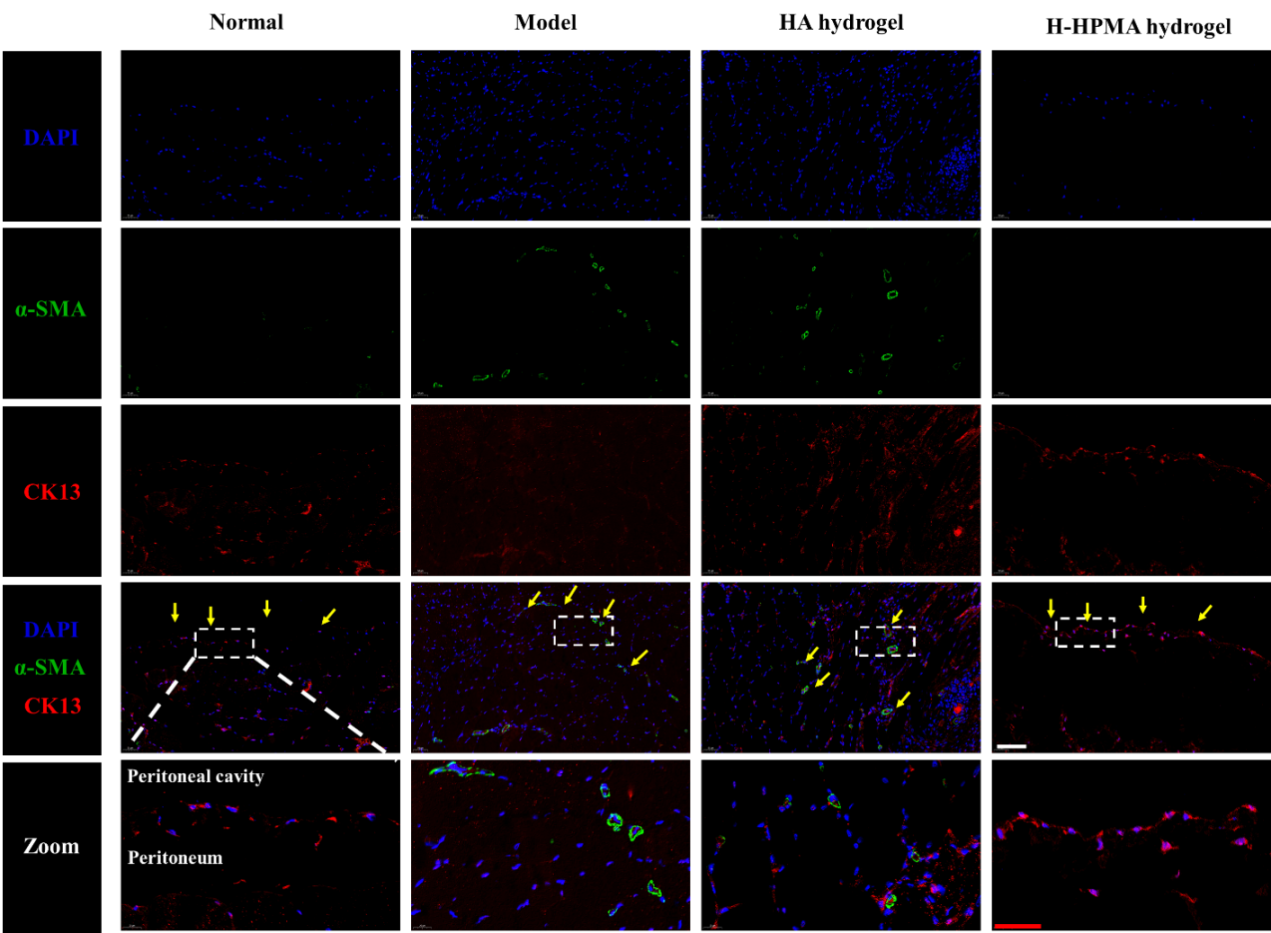


**Figure S13.** Representative immunofluorescence staining of different groups on day 5 after adhesiolysis. Sections of tissue were stained with DAPI (blue), CK13-Alexa Fluor®594 (red) and α-SMA-Alexa Fluor®488 (green). Magnification:200×; Scale bars represent 100 μm in the lower magnification images and 50 μm in the higher magnification images.


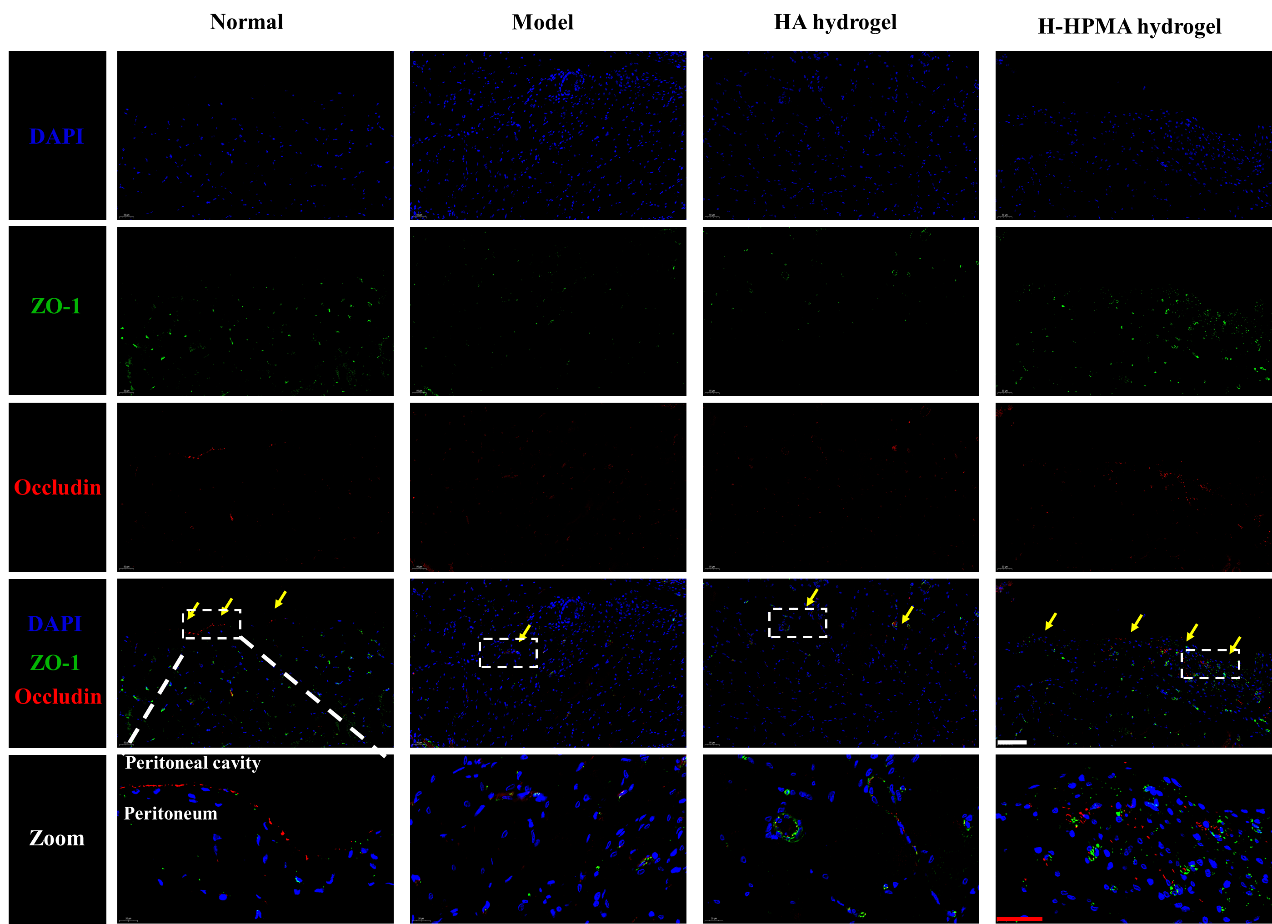


**Figure S14.** Images of immunofluorescence staining on day 5 after the first surgery. Sections of tissue were stained with DAPI (blue), occludin-Alexa Fluor®594 (red) and ZO-1-Alexa Fluor®488 (green). Magnification: 200×; Scale bars represent 100 μm in the lower magnification images and 50 μm in the higher magnification images.


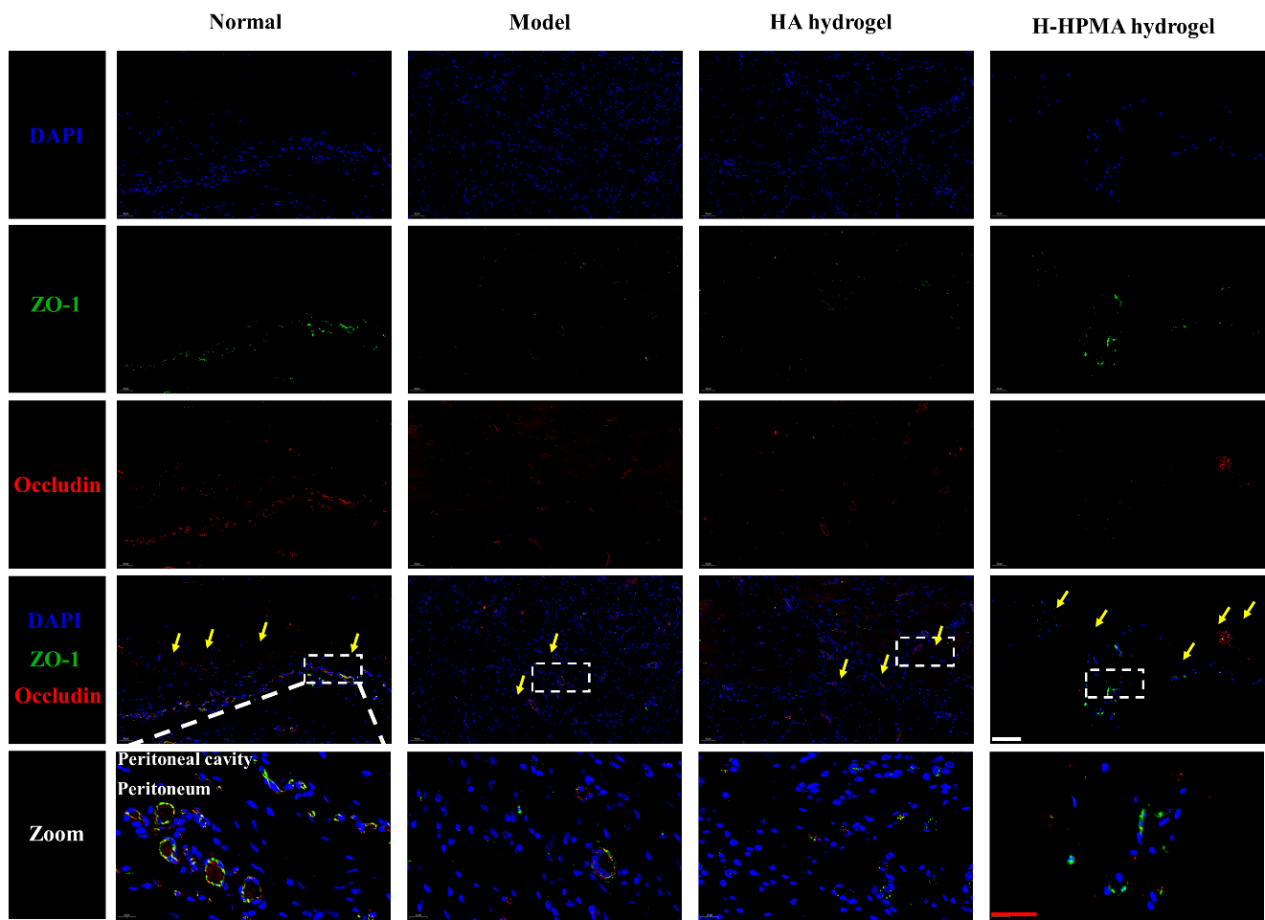


**Figure S15.** Images of immunofluorescence staining on day 5 after adhesiolysis. Sections of tissue were stained with DAPI (blue), occludin-Alexa Fluor® 594 (red) and ZO-1-Alexa Fluor®488 (green). Magnification: 200×; Scale bars represent 100 μm in the lower magnification images and 50 μm in the higher magnification images.

**
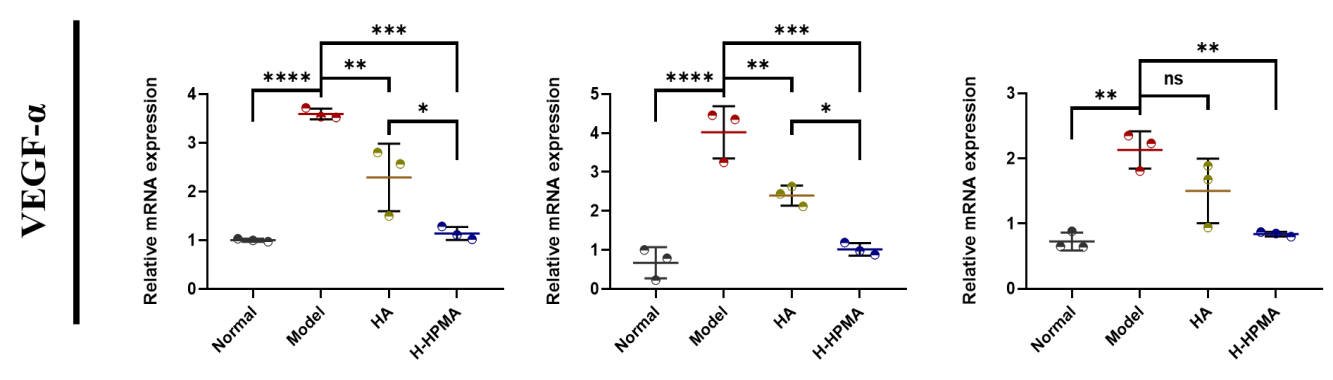
**

**Figure S16**. The VEGF-α expression were significantly up-regulated in model samples, compared with healthy adjacent peritoneal tissues. All data are presented as mean ± SD (n=3 per group); the ns means no significant difference; **p* < 0.05; ***p* < 0.01; ****p* < 0.001; *****p* < 0.0001.


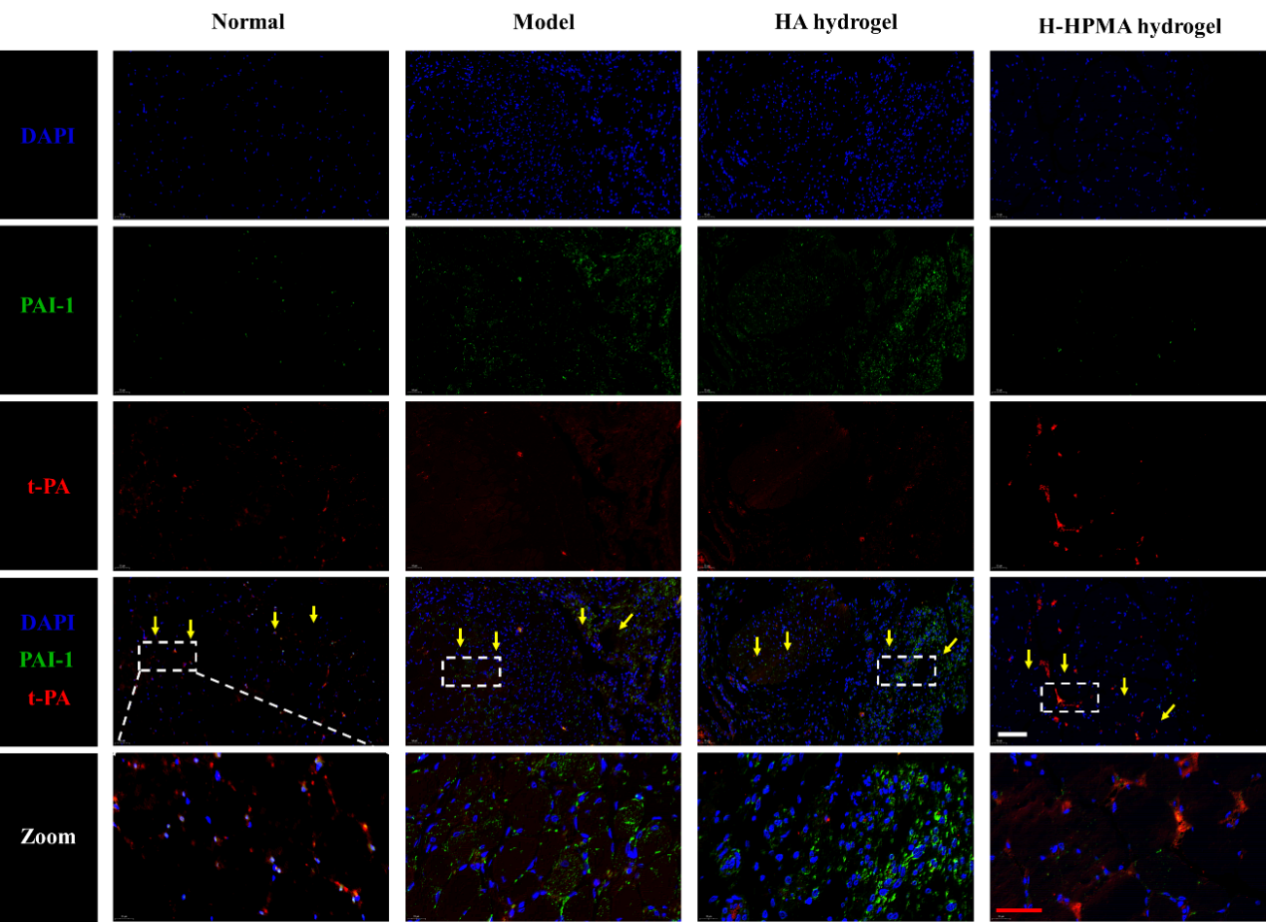


**Figure S17.** Images of immunofluorescence staining on day 5 after the first surgery. Sections of tissue were stained with DAPI (blue), t-PA-Alexa Fluor® 594 (red) and PAI-1-Alexa Fluor®488 (green). Magnification: 200×; Scale bars represent 100 μm in the lower magnification images and 50 μm in the higher magnification images.


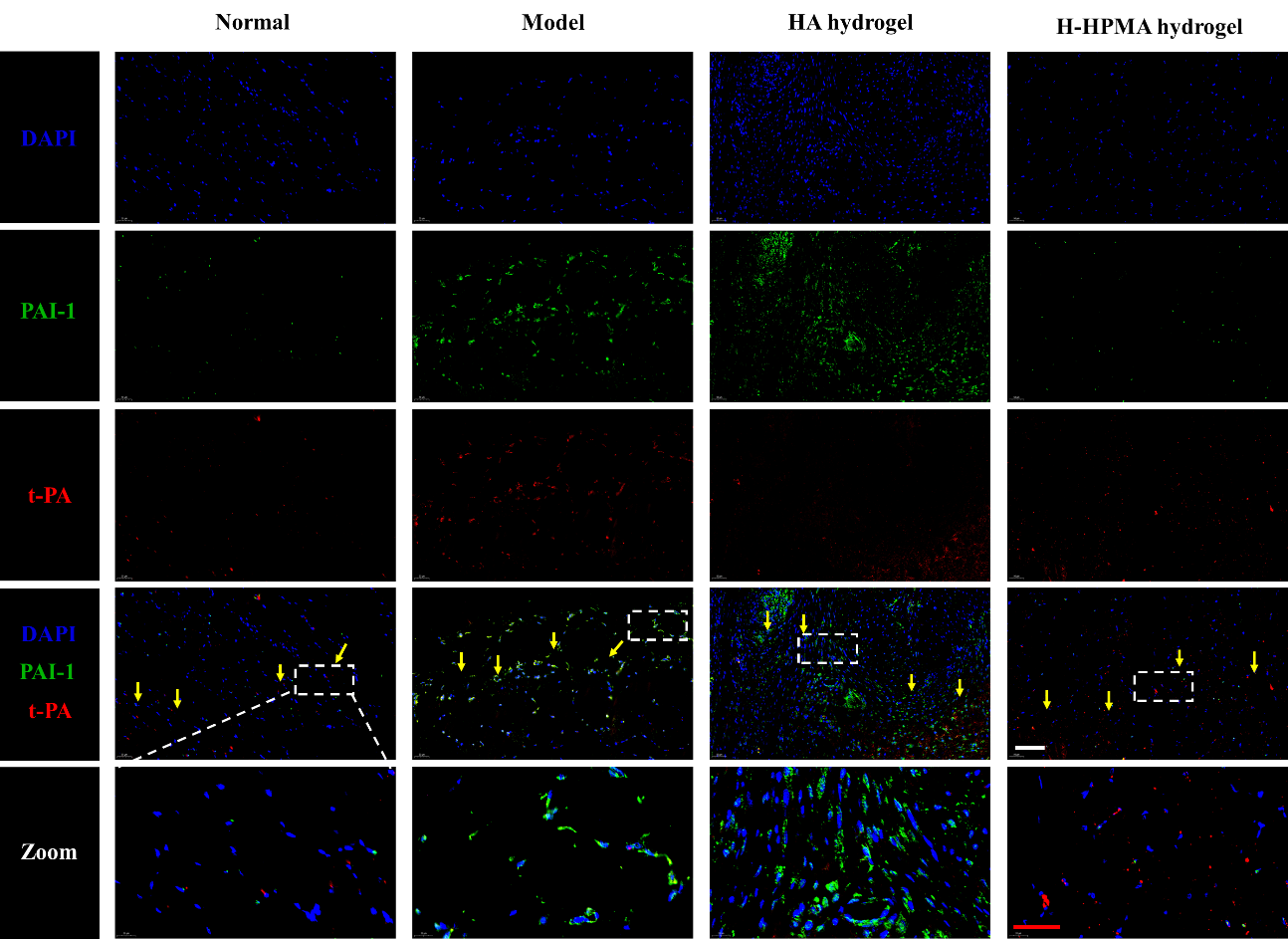


**Figure S18.** Images of immunofluorescence staining on day 5 after adhesiolysis. Sections of tissue were stained with DAPI (blue), t-PA-Alexa Fluor®594 (red) and PAI-1-Alexa Fluor®488 (green). Magnification: 200×; Scale bars represent 100 μm in the lower magnification images and 50 μm in the higher magnification images.


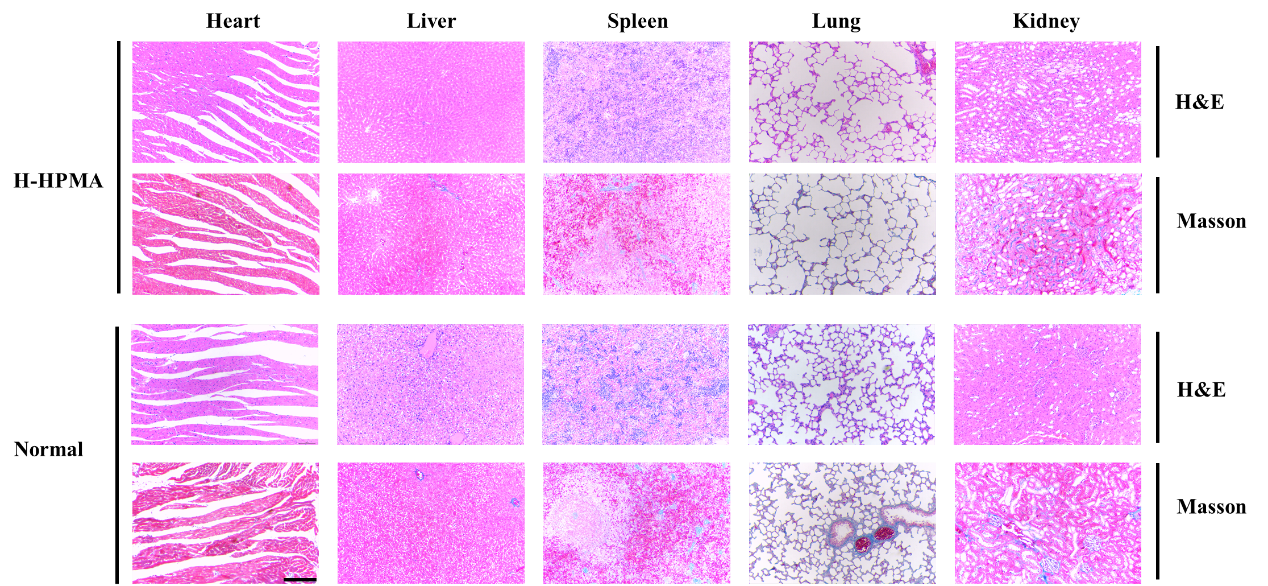


**Fig. S19.** H&E and Masson’s trichrome staining of major organs of rats in H-HPMA hydrogel therapy group. Magnification:40×; Scale bars: 400 μm.

**Table S1.** List of antibodies concentration used for western blotting

| **Antibody** | **Cat. no** | **Source** | **Dilution** |
| --- | --- | --- | --- |
| TGF-β1 | ab92486 | Abcam | 1:1000 |
| Smad3 | GTX108638 | GeneTex | 1:1000 |
| Smad7 | sc-365846 | Santa Cruz Biotechnology | 1:1000 |
| p65 | BS3157 | Bioworld | 1:1000 |
| GAPDH | ab8245 | Abcam | 1:5000 |

**Movie S1.** Extrusion of H-HPMA hydrogel from a syringe needle.
